# Recombinant chimeric horsepox virus (TNX-801) is attenuated relative to vaccinia virus strains in both *in vitro* and *in vivo* models

**DOI:** 10.1128/msphere.00265-24

**Published:** 2024-11-13

**Authors:** Stephanie V. Trefry, Mayanka Awasthi, Christy N. Raney, Amy L. Cregger, Chase A. Gonzales, Brittney L. Layton, Robert N. Enamorado, Nelson A. Martinez, Deborah S. Gohegan, Masoudeh Masoud-Bahnamiri, Jennifer Y. Cho, Dawn M. Myscofski, Tinoush Moulaei, Natasza E. Ziółkowska, Scott J. Goebel, Seth Lederman, Sina Bavari, Farooq Nasar

**Affiliations:** 1 Discovery and Translations Sciences, Tonix Pharmaceuticals, Frederick, Maryland, USA; University of Saskatchewan, Saskatoon, Saskatchewan, Canada

**Keywords:** *Orthopoxvirus*, horsepox, vaccinia, immunocompromised mice, human primary cells

## Abstract

Recombinant chimeric horsepox virus (TNX-801) is a preclinical vaccine in development against mpox and smallpox. In this report, we investigated the potential phenotypic differences in *in vitro* and *in vivo* models between TNX-801 and older vaccinia virus (VACV)-based vaccine strains (VACV-Lis and VACV-NYCBH) used in the eradication of smallpox as well as VACV-WR, VACV-IHD, and MVA. TNX-801 displayed a small plaque phenotype (~1–2 mm) in BSC-40 and Vero-E6 cells. Multi-step replication kinetics in immortalized nonhuman primate cell lines, and human primary cells from dermal and respiratory tracts yielded >10- to 100-fold lower infectious titers than the VACV strains. In addition, the infectious particle-to-genome copy ratio data suggests that TNX-801 genome packaging is ~10- to 100-fold less efficient than the VACV strains and the potential mechanism of TNX-801 attenuation is at the packaging/egress stage. Lastly, the susceptibility to VACV and TNX-801 infection of three new immunocompromised murine models (C56BL/6 *Ifnar*^−/−^, C56BL/6 *Ifngr*^−/−^, and C56BL/6 *Ifnar*^−/−^/*Ifngr*^−/−^) was investigated. VACV strains were able to produce severe disease including decrease in body weight and temperature, as well as lethality in murine models via the intraperitoneal or intranasal routes. In contrast to VACV strains, TNX-801 was unable to produce any disease in murine models. These data demonstrate that TNX-801 is >10- to 1,000-fold more attenuated compared to older VACV-based smallpox vaccine strains in human primary cell lines and immunocompromised mice.

**IMPORTANCE:**

Variola and monkeypox viruses are medically important pathogens that can cause fatal human disease. The two FDA-approved vaccines, ACAM-2000 and JYNNEOS, have important advantages and disadvantages. ACAM-2000 offers durable immunity; however, it has high adverse event rates. In contrast, JYNNEOS has a safer profile but requires two doses 4-weeks apart to achieve comparable immunity. Consequently, there is a need for vaccines offering durable immunity via single immunization with minimal adverse events. TNX-801 is a preclinical stage vaccine that can stimulate potent immunity via a single dose and provides protection against lethal mpox disease in the nonhuman primate model. Here, we show that TNX-801 is >10- to 1,000-fold attenuated in *in vitro* and *in vivo* models including human primary cells and immunocompromised murine models than vaccine strains utilized in smallpox eradication. The natural attenuation of TNX-801 and its ability to induce protective immunity via a single vaccination are promising and warrants further development.

## INTRODUCTION

The family *Poxviridae* is comprised of viruses with linear double-stranded DNA genomes with covalently enclosed ends, ~128 to ~456 kb in length (1–4). The virions are brick-shaped and ~220 to 450 nm long × 140 to 260 nm wide × 140 to 260 nm thick. The family includes two subfamilies and 22 genera with members that can infect vertebrates or invertebrates. Of these viruses, two members of the genus *Orthopoxvirus*, variola and monkeypox, are medically important pathogens as human infection can produce fatal disease. Case fatality rates for variola, the causative agent of smallpox disease, and monkeypox viruses range from ~30 to 50% and ~1 to 11%, respectively (4).

Smallpox has been a significant human pathogen throughout history with the earliest known written records and variola virus sequences dating back to ~1,570 B.C.E. and ~600 C.E., respectively (4, 5). Prior to eradication, smallpox killed ~250 million people during the 20th century (4, 6, 7). The smallpox vaccine effort was the product of Edward Jenner’s seminal observation in milkmaids who contracted a mild disease from cows and were subsequently protected from lethal smallpox. For 143 years following his observation, the agent in the vaccine was thought to be of cowpox origin (4, 6, 7). However, in 1939, it was shown that the vaccine was comprised of vaccinia virus (VACV), a serologically related virus but distinct from cowpox (8). The origin of VACV, its natural cycle, and how it was introduced into the smallpox vaccine remains unknown. Nonetheless, following the discovery of VACV, multiple virus strains (New York City Board of Health [NYCBH], Lister [Lis], and others) were developed and utilized as smallpox vaccines throughout the world eventually leading to eradication by 1980 (4, 9–11). Currently, the variola virus only exists at the CDC (USA) and Vector Labs (Russia). In the decades following eradication, two new vaccines (ACAM-2000 and JYNNEOS) were developed in the event of bioterror and/or accidental release of smallpox (9, 11–17). ACAM-2000 is a live, replication-competent vaccine derived by plaque picking VACV variants from the older Dryvax vaccine, both derived from VACV-NYCBH. JYNNEOS (also known as Imvamune or Imvanex) comprises modified vaccinia Ankara (MVA), a nonreplicating virus generated by serial passaging in primary chicken cells.

Monkeypox virus (MPXV) is closely related to variola virus; however, it causes similar but clinically milder disease in humans, referred to as mpox (4, 18). The virus was discovered in 1958 in non-human primate colonies, and the first reported human case was in 1970 in Zaire (now the Democratic Republic of the Congo) (19–23). In the following five decades, sporadic human outbreaks were reported in West and Central Africa. However, in 2022, the largest recorded outbreak of mpox occurred which rapidly spread to 116 countries with ~93,000 reported cases and 170 deaths (24). Currently, the only FDA-approved vaccine against mpox is JYNNEOS, which has been utilized to curtail the 2022 outbreak (25, 26).

The older (VACV-NYCBH, VACV-Lis) as well as the new (ACAM-2000 and JYNNEOS) vaccines have important advantages and disadvantages. The older VACV vaccines and ACAM-2000 induced durable immunity via a single immunization; however, these vaccines also cause severe adverse events including pericarditis, myocarditis, encephalitis, and death (9, 11, 13, 27–33). The new JYNNEOS vaccine has a superior safety profile; however, it requires two doses 4-weeks apart for optimal immunity (25, 26). Due to these reasons, there is a critical need to develop vaccines capable of inducing durable immunity via a single vaccination with minimal adverse events.

**Fig 1:**
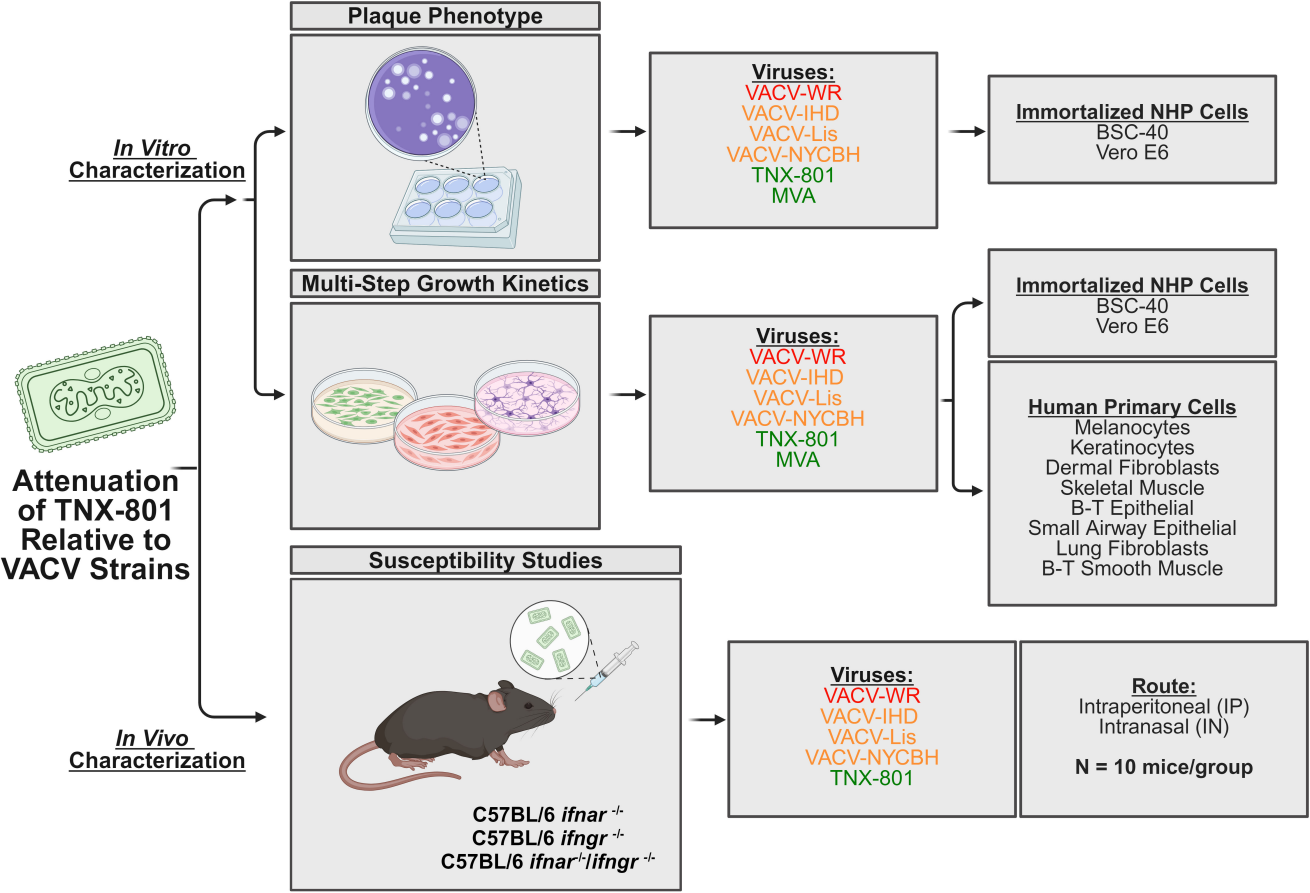
Experimental design of *in vitro* and *in vivo* studies investigating the attenuation of TNX-801 relative to VACV vaccine strains.

To address the need for new vaccines, we investigated another member of the genus *Orthopoxvirus*, Horsepox virus (HPXV), as a potential vaccine against mpox. Utilizing modern genetic techniques, we generated an infectious HPXV clone derived from the MNR-76 (TNX-801) isolate and investigated its immunogenicity and efficacy (34–36). A single TNX-801 vaccination at either ~6.6 or 5.7 log_10_ PFU via scarification route induced humoral responses and was able to protect nonhuman primates (NHPs) from a lethal MPXV challenge (35). Following the promising efficacy data in NHPs, we examined the next critical hurdle by investigating the potential attenuation of TNX-801. This study examined the attenuation of TNX-801 in immortalized NHP cell lines, primary human cells, and immunocompromised murine models relative to the older vaccinia virus-based vaccine strains (VACV-Lister and VACV-NYCBH) utilized to eradicate smallpox as well as mouse-adapted virulent VACV strains (VACV-WR and VACV-IHD) and MVA (basis of JYNNEOS vaccine) (Fig. 1).

## RESULTS

### TNX-801 plaque phenotype

**Fig 2:**
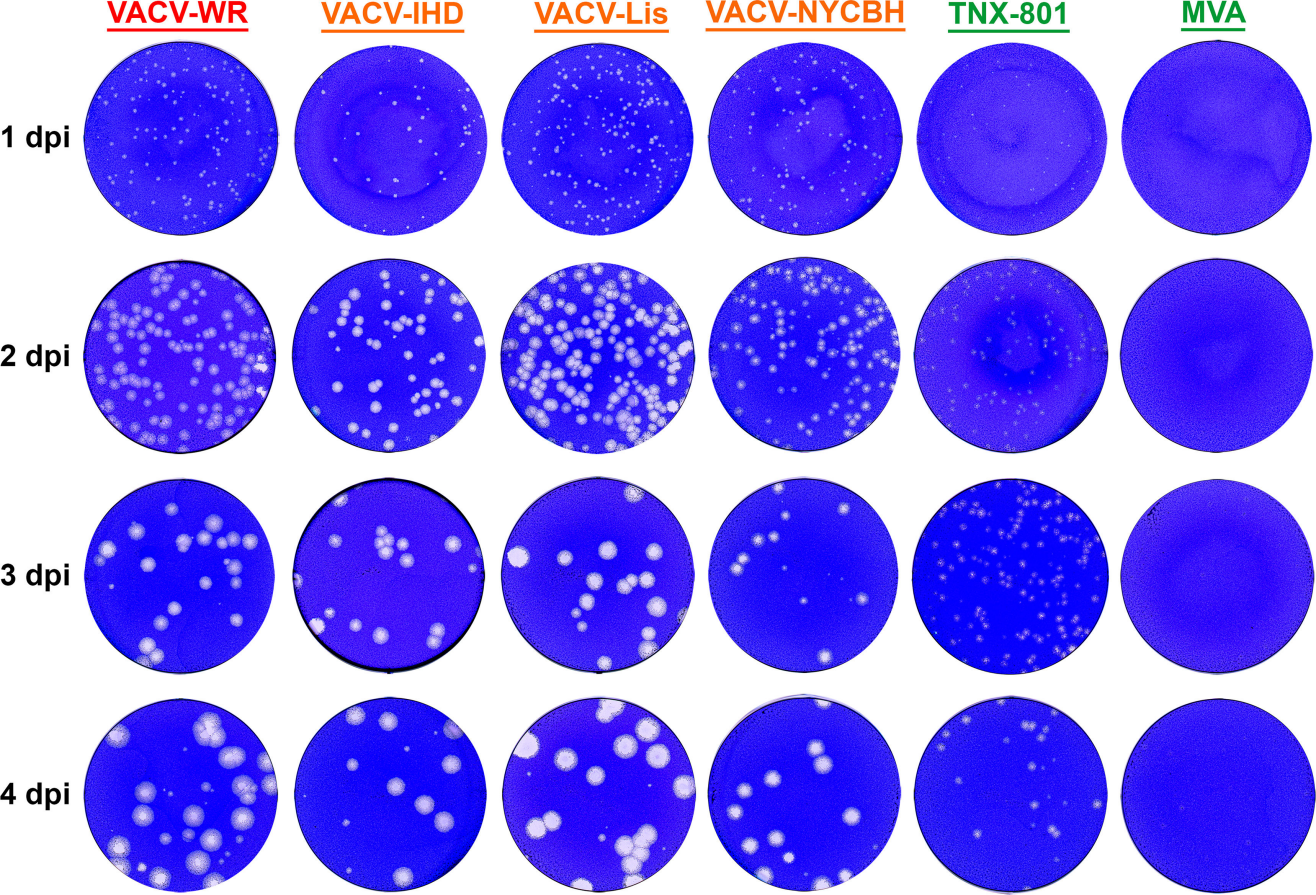
Plaque phenotype of VACV-WR, VACV-IHD, VACV-Lis, VACV-NYCBH, TNX-801, and MVA in BSC-40 cells. Representative wells from six-well plates each day are shown in photographs.

**Fig 3:**
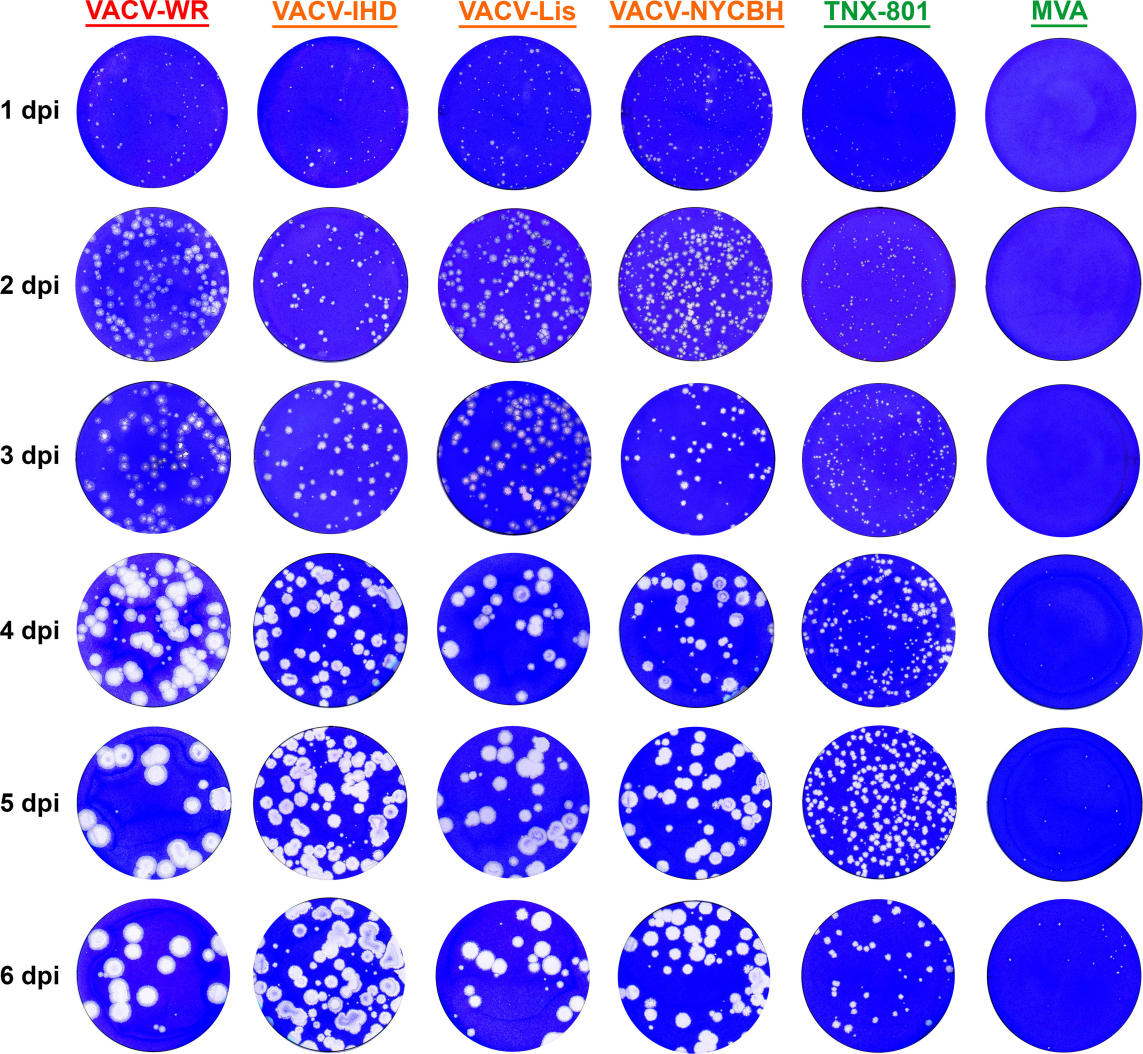
Plaque phenotype of VACV-WR, VACV-IHD, VACV-Lis, VACV-NYCBH, TNX-801, and MVA in Vero-E6 cells. Representative wells from six-well plates each day are shown in photographs.

The plaque phenotype of TNX-801 was investigated relative to VACV-WR, VACV-IHD, VACV-Lis, VACV-NYCBH, and MVA on immortalized NHP cell lines, BSC-40 and Vero-E6 (Fig. 1 to 3). In both cell lines, VACV strains displayed plaques starting at 1 day-post-infection (dpi) and yielded a large plaque phenotype ~3–4 mm in diameter by 4 (BSC-40) and 6 (Vero-E6) dpi. In contrast, TNX-801 and MVA displayed a small plaque phenotype, ~1–2 mm and ~0.5 mm in diameter on both cell lines, respectively.

### Multi-step replication kinetics in NHP cell lines and human primary cells

**Fig 4:**
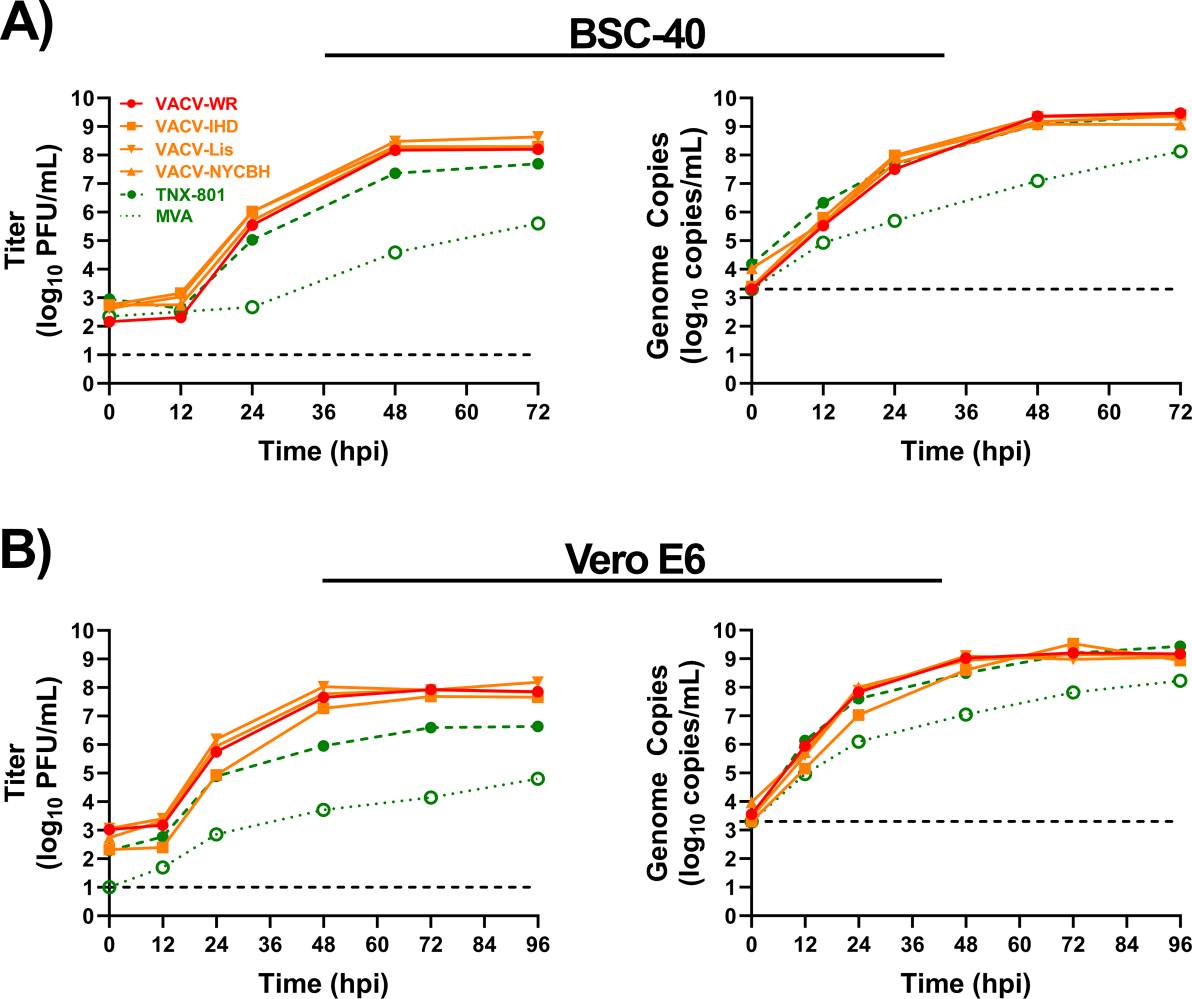
Multi-step growth kinetics of VACV-WR, VACV-IHD, VACV-Lis, VACV-NYCBH, TNX-801, and MVA in BSC-40 (**A**) and Vero-E6 (**B**) cells at an MOI of 0.01. Samples were taken at 0, 12, 24, 48, 72, and 96 hpi (Vero-E6 only). Triplicate six-wells were sampled at each time-point followed by three cycles of freeze/thaw/sonication and titrated on BSC-40 cells. The limit of detection is shown for the infectious (1.0 log_10_ PFU/mL) and qPCR (3.3 log_10_ genome copies/mL) assays with black dashed lines.

The replication kinetics of VACV strains, TNX-801, and MVA were investigated in BSC-40 and Vero-E6 cells at an multiplicity of infection (MOI) of 0.01 (Fig. 4A and B). All VACV strains reached peak titers of ~8.0 log_10_ PFU/mL and ~9.0 log_10_ genome copies/mL between 48 and 72 hpi. In contrast, TNX-801 and MVA displayed delayed replication kinetics by 24 hrs and achieved peak titers between 72 and 96 hpi. The peak infectious titer of TNX-801 was ~7.7 and ~6.6 log_10_ PFU/mL in BSC-40 and Vero-E6 cells, respectively. The peak genome copy values in both cell lines were ~9.0 log_10_ genome copies/mL. Although the peak genome copy was comparable between VACV strains and TNX-801, the peak infectious titers of TNX-801 were up to ~10- to 119-fold lower (Fig. 4A and B). MVA infection yielded peak titers of ~5.6 and ~4.8 log_10_ PFU/mL in BSC-40 and Vero-E6 cells, respectively. The peak genome copy values in both cell lines were ~8.0 log_10_ genome copies/mL.

**Fig 5:**
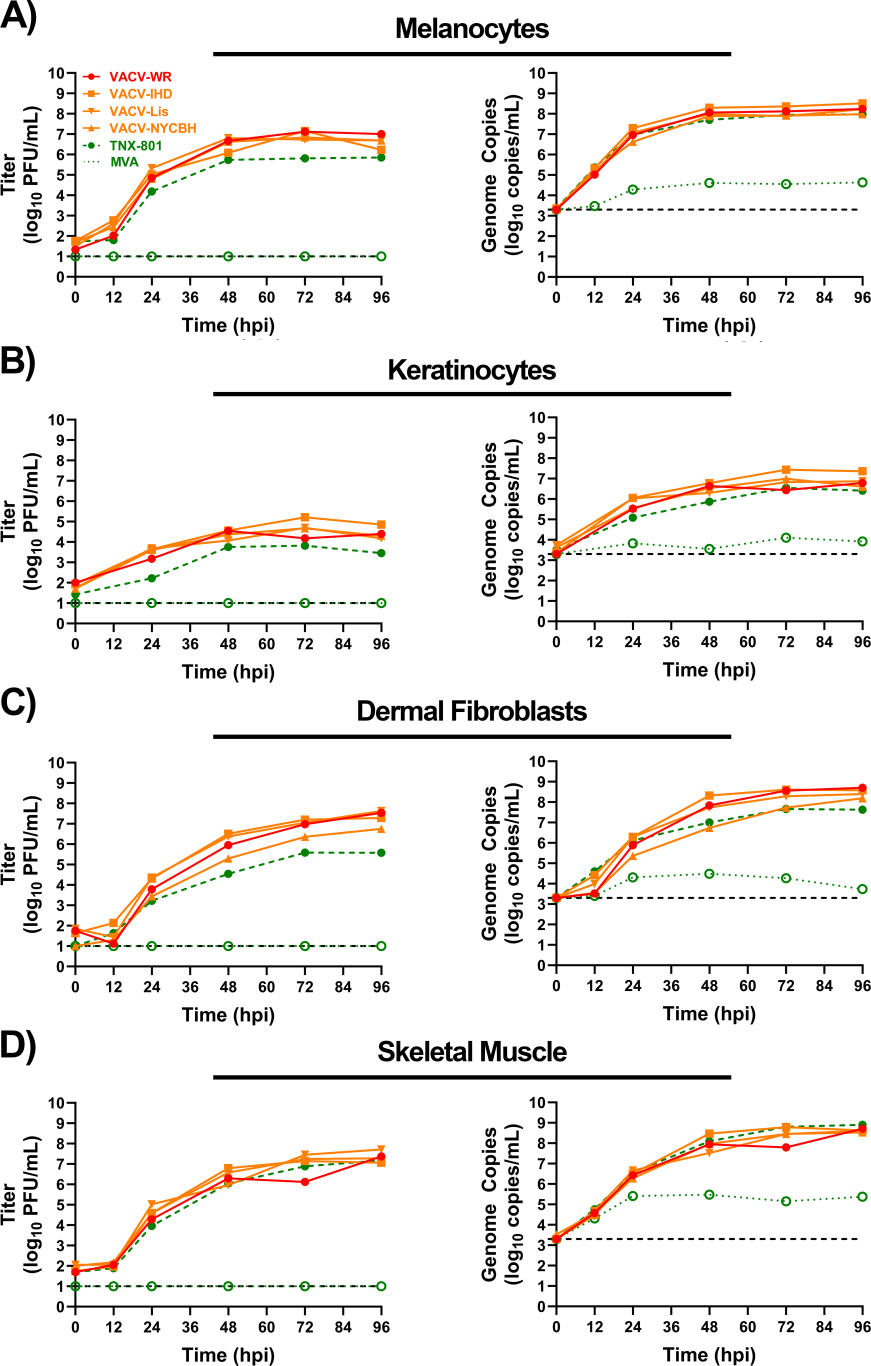
Multi-step growth kinetics of VACV-WR, VACV-IHD, VACV-Lis, VACV-NYCBH, TNX-801, and MVA in primary human cell lines from the dermal tract (melanocytes [**A**], keratinocytes [**B**], fibroblasts [**C**], and skeletal muscle cells [**D**]) at an MOI of 0.01. Samples were taken at 0, 12, 24, 48, 72, and 96. Triplicate six-wells were sampled at each time-point followed by three cycles of freeze/thaw/sonication and titrated on BSC-40 cells. The limit of detection is shown for the infectious (1.0 log_10_ PFU/mL) and qPCR (3.3 log_10_ genome copies/mL) assays with black dashed lines.

Multi-step replication studies at an MOI of 0.01 were also performed in human primary cells from the dermal tract (melanocytes, keratinocytes, dermal fibroblasts, and skeletal muscle) (Fig. 5). Infection with VACV strains and TNX-801 of primary melanocytes, keratinocytes, and fibroblasts displayed similar replication kinetics (Fig. 5A through C). The peak infectious titers of VACV strains and TNX-801 ranged from ~4.5 to 7.5 log_10_ PFU/mL and ~3.8 to 5.9 in log_10_ PFU/mL, respectively. The peak infectious titers of TNX-801 were up to ~5- to 112-fold lower in all three primary cell lines, whereas the peak genome copy values of VACV strains and TNX-801 were comparable, ~6.7 to 9.0 log_10_ genome copies/mL. In contrast, both TNX-801 and VACV strains replicated to similar levels in skeletal muscle cells with peak titers and genome copies of ~7.3 log_10_ PFU/mL and ~8.9 log_10_ genome copies/mL, respectively (Fig. 5D). MVA was unable to produce infectious virus in all cells throughout the 96-hr sampling period. However, genome copies were detected throughout, with peak values of ~4.1 to 5.5 log_10_ genome copies/mL at 48 to 72 hpi.

**Fig 6:**
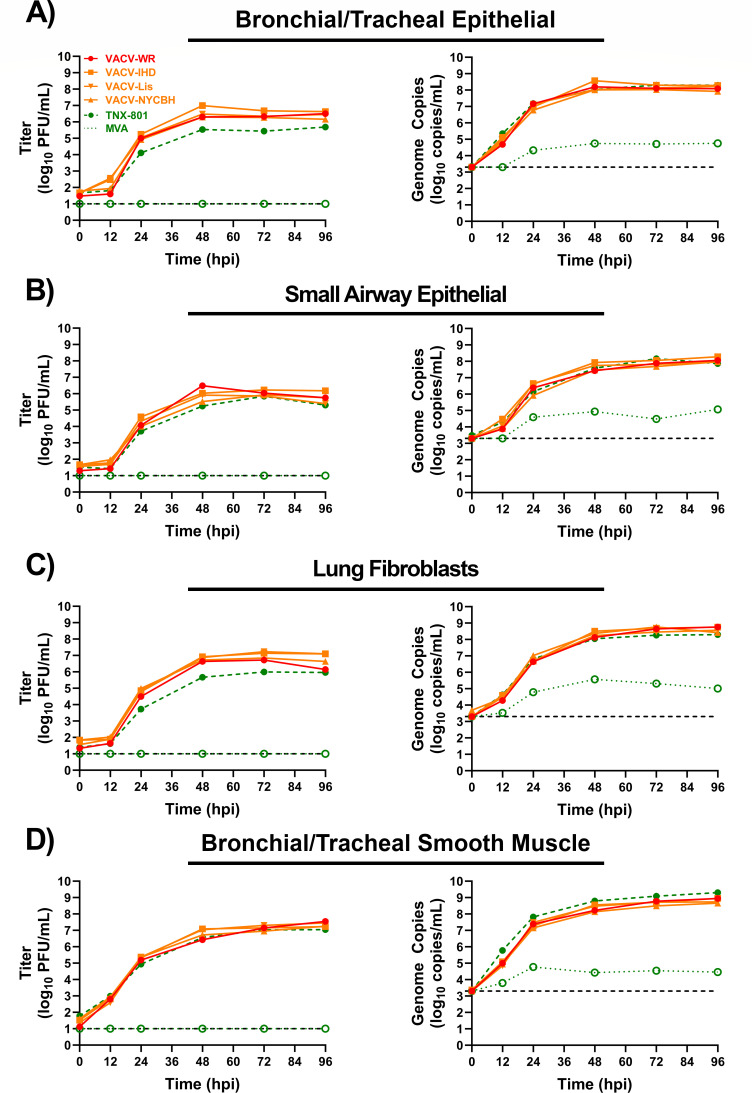
Multi-step growth kinetics of VACV-WR, VACV-IHD, VACV-Lis, VACV-NYCBH, TNX-801, and MVA in primary human cell lines from the respiratory tract (bronchial/tracheal epithelial cells [**A**], small airway epithelial cells [**B**], lung fibroblasts [**C**], and bronchial/tracheal smooth muscle [**D**]) at an MOI of 0.01. Samples were taken at 0, 12, 24, 48, 72, and 96 hpi. Triplicate six-wells were sampled at each time-point followed by three cycles of freeze/thaw/sonication and titrated on BSC-40 cells. The limit of detection is shown for the infectious (1.0 log_10_ PFU/mL) and qPCR (3.3 log_10_ genome copies/mL) assays with black dashed lines.

The infection of primary cells from the respiratory tract (bronchial/tracheal epithelial, small airway epithelial, lung fibroblasts, and bronchial/tracheal smooth muscle) yielded similar results to the dermal tract (Fig. 6). VACV infection of fibroblasts, bronchial/tracheal epithelial, and small airway epithelial cells yielded peak titers of ~5.9 to 7.2 log_10_ PFU/mL and ~8.0 to 8.8 log_10_ genome copies/mL, respectively (Fig. 6A through C). TNX-801 produced peak infectious titers and genome copies ranging from ~5.7 to 6.0 log_10_ PFU/mL and ~8.3 log_10_ genome copies/mL, respectively (Fig. 6A through C). Despite comparable levels of genome copy between VACV strains and TNX-801, the TNX-801 infectious titers were up to ~5- to 28-fold lower than VACV strains in all three cell lines. In contrast to the other primary cells, TNX-801 and VACV strains replicated to comparable levels in bronchial/tracheal smooth muscle cells with peak titers of ~7.0 to 7.5 log_10_ PFU/mL and peak genome copy of ~8.7 to 9.3 log_10_ genome copies/mL (Fig. 6D). Lastly, MVA infection did not produce any infectious virus; however, genome copies were detected in cells throughout the infection with peak values of ~4.8 to 5.6 log_10_ genome copies/mL at 48 to 96 hpi.

To investigate the potential mechanism of TNX-801 attenuation *in vitro*, the infectious particle-to-genome copy ratios in NHP cell lines and human primary cells were calculated (Table 1; Fig. S1 to S10). The infectious particle-to-genome copy ratios for VACV strains ranged from ~1:5 to 1:17 and ~1:16 to 1:56 in BSC-40 and Vero-E6 cells, respectively. In contrast, TNX-801 and MVA ratios in BSC-40 and Vero-E6 were ~1:52 to 1:390 and ~1:336 to 1:4,023, respectively. VACV strains yielded ratios ranging from ~1:9 to 1:192 in human primary cell lines. In contrast, TNX-801 yielded consistently higher ratios than VACV ranging from ~1:66 to 1:504. The MVA ratios in human primary cell lines were not calculated as MVA did not produce infectious virus.

**TABLE 1:** Infectious particle to genome ratios of VACV strains, TNX-801, and MVA in immortalized and human primary cell lines^*a*^

Cell line	VACV-WR	VACV-IHD	VACV-Lis	VACV-NYCBH	TNX-801	MVA
BSC-40	1:17	1:12	1:5	1:7	1:52	1:336
Vero-E6	1:20	1:56	1:12	1:16	1:390	1:4,023
Melanocytes	1:14	1:28	1:15	1:14	1:116	*−*
Keratinocytes	1:142	1:169	1:142	1:192	1:345	*−*
Dermal fibroblasts	1:21	1:26	1:9	1:26	1:124	*−*
Skeletal muscle	1:25	1:42	1:9	1:18	1:66	*−*
BT epithelial	1:69	1:39	1:47	1:55	1:504	*−*
SA epithelial	1:25	1:72	1:78	1:67	1:208	*−*
Lung fibroblast	1:32	1:39	1:24	1:46	1:246	*−*
BT smooth muscle	1:32	1:41	1:23	1:28	1:152	*−*

^*a*^ The ratios were generated at peak virus output by dividing infectious titers by genome copy. MVA ratios in human primary cell lines were not calculated as MVA infection did not lead to the production of infectious virus. "−” indicates not performed.

### Investigating infection of VACV strains and TNX-801 in C56BL/6 *Ifnar*^−/−^ and C56BL/6 *Ifngr*^−/−^ mice via the intraperitoneal route

**Fig 7:**
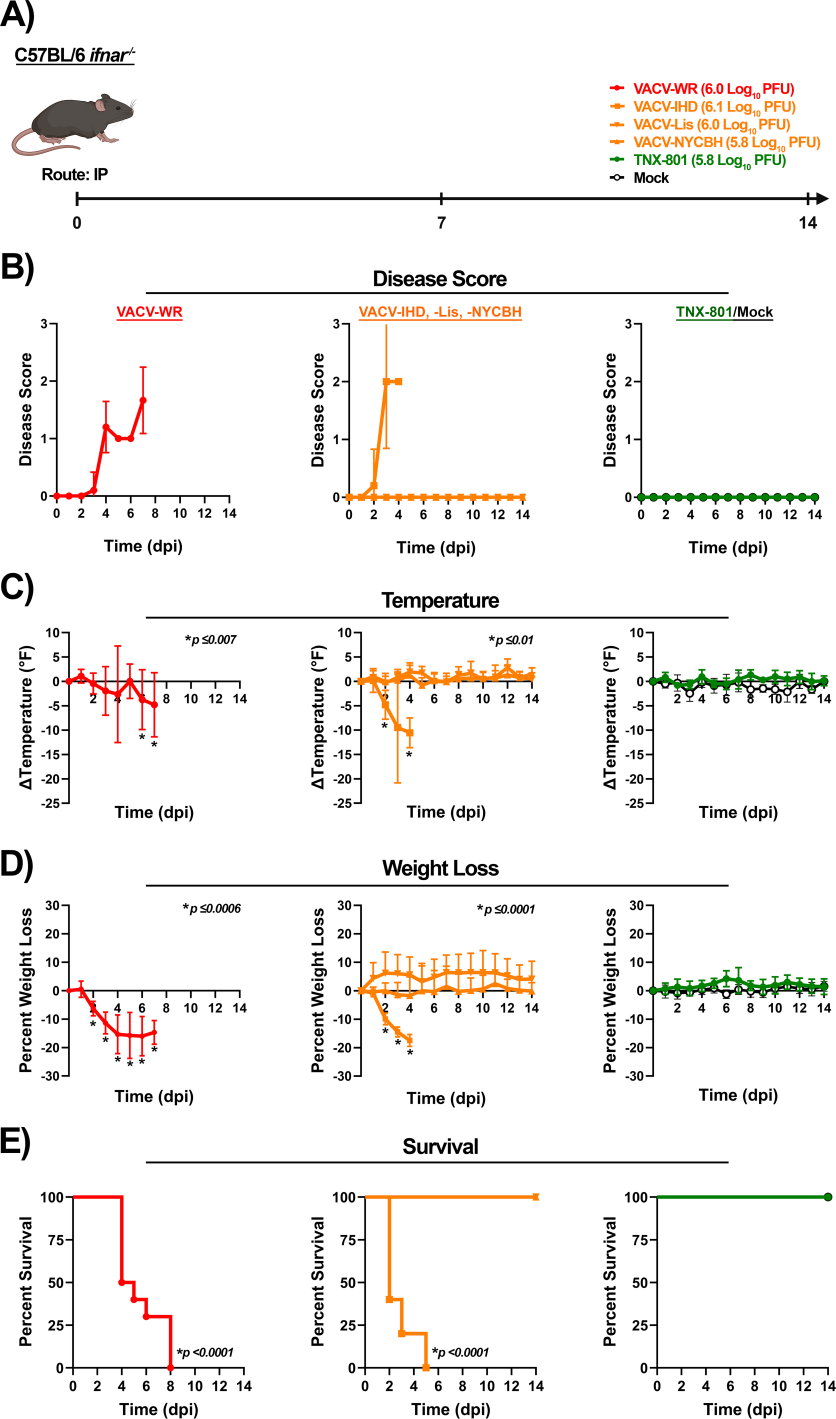
Infection of C57BL/6 *Ifnar^−/−^* mice at 6.0 log_10_ PFU/mouse dose with VACV-WR, VACV-IHD, VACV-Lis, VACV-NYCBH, TNX-801, and phosphate-buffered saline (PBS) via intraperitoneal (IP) route (**A**). Following infection, mice were monitored for disease score (**B**), body temperature (**C**), weight loss (**D**), and survival (**E**). The target dose for each virus was verified via the plaque assay, and values are provided (**A**). *p*-values comparing VACV strains to TNX-801 and mock groups are provided in each graph.

**Fig 8:**
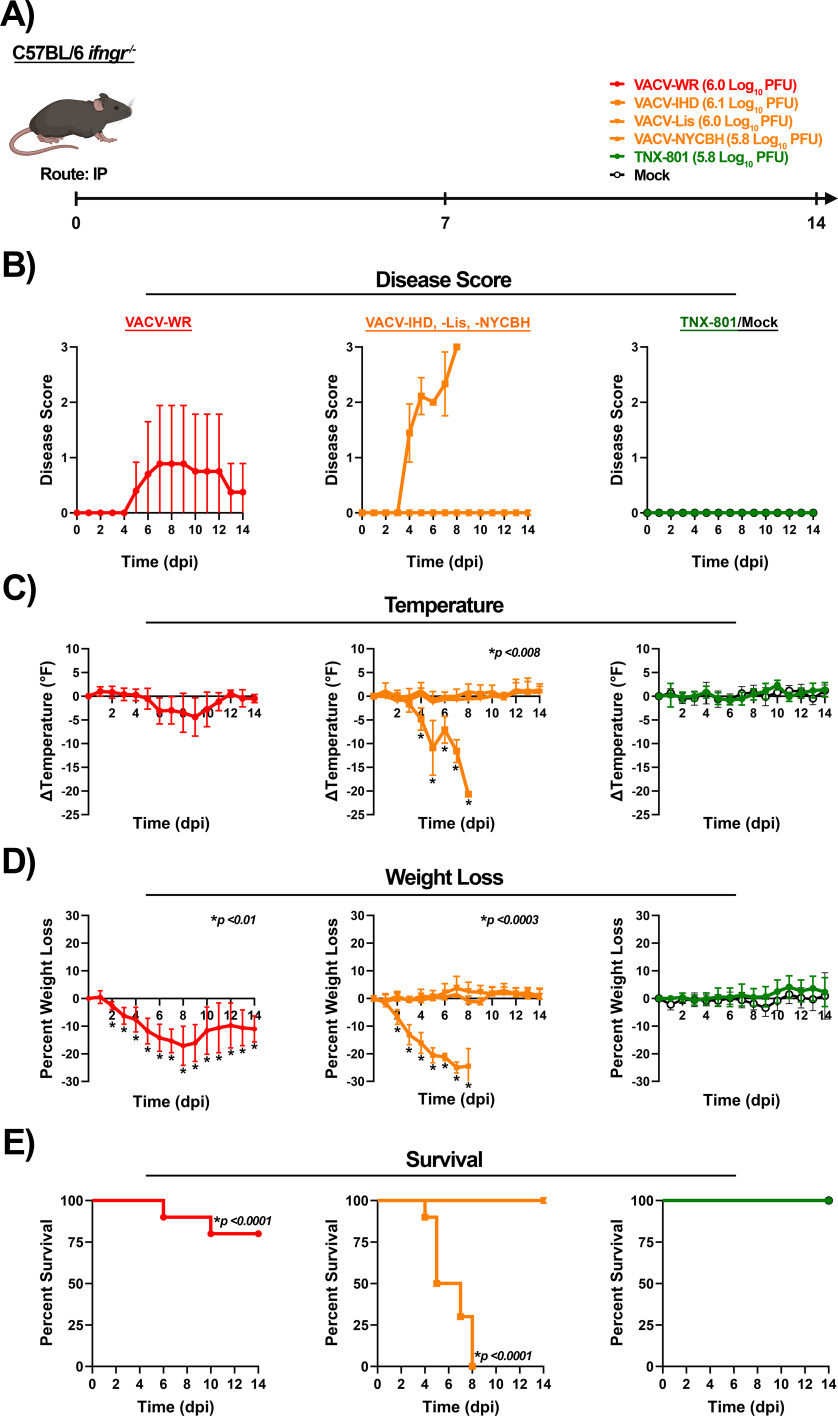
Infection of C57BL/6 *Ifngr^−/−^* mice at 6.0 log_10_ PFU/mouse dose with VACV-WR, VACV-IHD, VACV-Lis, VACV-NYCBH, TNX-801, and PBS via intraperitoneal (IP) route (**A**). Following infection, mice were monitored for disease score (**B**), body temperature (**C**), weight loss (**D**), and survival (**E**). The target dose for each virus was verified via the plaque assay, and values are provided (**A**). *p*-values comparing VACV strains to TNX-801 and mock groups are provided in each graph.

C56BL/6 *Ifnar*^−/−^ and C56BL/6 *Ifngr*^−/−^ mice were infected with VACV-WR, VACV-IHD, VACV-Lis, VACV-NYCBH, and TNX-801 at 6.0 log_10_ PFU/mouse dose via the intraperitoneal (IP) route (Fig. 7A and 8A). Following the infection of C56BL/6 *Ifnar*^−/−^ mice, VACV-WR and VACV-IHD groups exhibited severe disease at 2 to 3 dpi with a peak disease score of ~2 at 3 dpi (VACV-IHD) and ~1.7 at 7 dpi (VACV-WR) (Fig. 7B). Body temperature began to decline as early as 2 dpi with peak decline at 4 (VACV-IHD) and 7 (VACV-WR) dpi of ~−4.8 to −10.5°F (Fig. 7C). Body weight declined by 2 dpi with peak weight loss at 4 (VACV-IHD) and 6 (VACV-WR) dpi of ~−16% to −17.5%, and all mice met the euthanasia criteria by 2 to 8 dpi (Fig. 7D and E). In contrast, VACV-Lis, VACV-NYCBH, TNX-801, and mock-infected mice did not exhibit any disease (Fig. 7B through E).

The infection of C56BL/6 *Ifngr*^−/−^ mice with VACV strains exhibited a similar disease pattern as C56BL/6 *Ifnar*^−/−^ mice. VACV-IHD-infected mice exhibited considerably more severe disease than VACV-WR-infected mice (Fig. 8B). VACV-WR- and VACV-IHD-infected mice exhibited disease between 4 and 5 dpi with peak disease scores of ~1 to 3 by 7 to 8 dpi (Fig. 8B). Body temperature declined between 3 and 6 dpi with peak decline of ~−20.7°F at 8 dpi (VACV-IHD) and ~−4.3°F at 9 dpi (VACV-WR) (Fig. 8C). Body weight declined starting at 2 dpi with peak weight loss between 7 to 8 dpi of ~−25% (VACV-IHD) and ~−17% (VACV-WR) (Fig. 8D). All VACV-IHD and 20% of VACV-WR mice met the euthanasia criteria between 4 and 10 dpi (Fig. 8E). In contrast, VACV-Lis, VACV-NYCBH, TNX-801, and mock-infected mice did not exhibit any disease (Fig. 8B–E).

### Investigating infection of VACV strains and TNX-801 in C56BL/6 *Ifnar*^−/−^ and C56BL/6 *Ifngr*^−/−^ mice via the intranasal route

**Fig 9:**
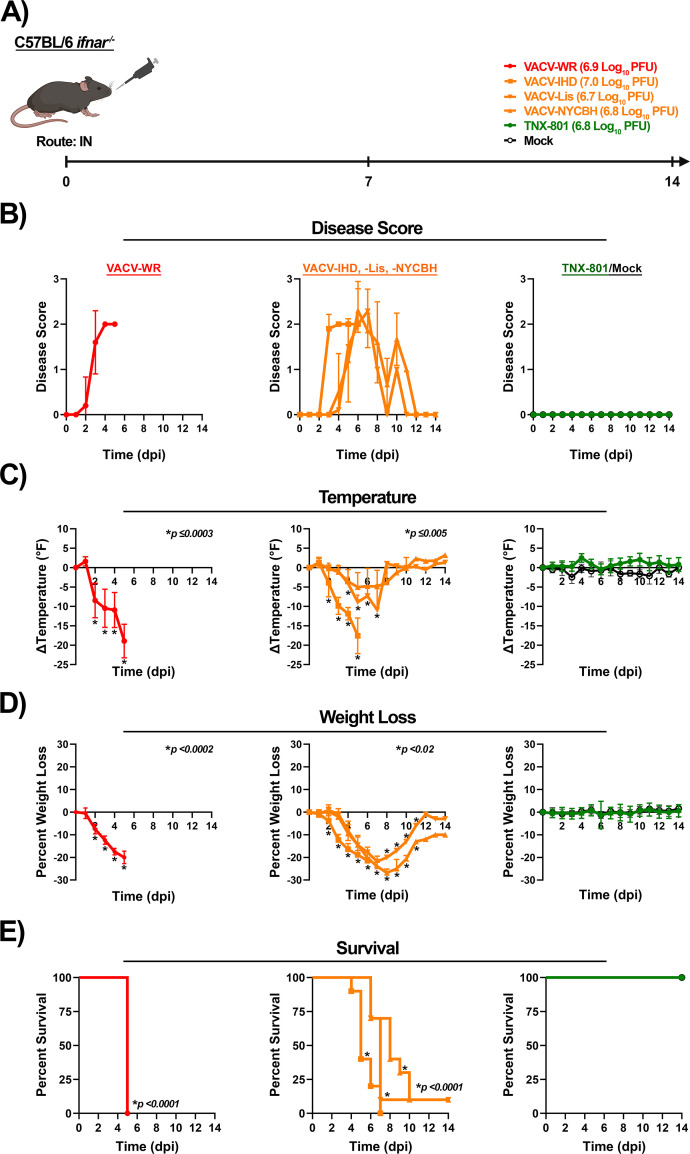
Infection of C57BL/6 *Ifnar^−/−^* mice at 7.0 log_10_ PFU/mouse dose with VACV-WR, VACV-IHD, VACV-Lis, VACV-NYCBH, TNX-801, and PBS via intranasal (IN) route (**A**). Following infection, mice were monitored for disease score (**B**), body temperature (**C**), weight loss (**D**), and survival (**E**). The target dose for each virus was verified via the plaque assay, and values are provided (**A**). *p*-values comparing VACV strains to TNX-801 and mock groups are provided in each graph.

The susceptibility of C56BL/6 *Ifnar*^−/−^ mice to VACV and TNX-801 infection via the intranasal (IN) route was investigated at 7.0 log_10_ PFU/mouse (Fig. 9A). Mice infected with VACV strains rapidly exhibited severe disease (Fig. 9). VACV-WR- and VACV-IHD-infected mice exhibited disease starting at 2 to 3 dpi with alterations in disease scores, body temperature, and weight (Fig. 9B through D). Mice exhibited peak disease scores of ~2 at 4 to 6 dpi and a decline in body temperature of ~−19°F at 5 dpi (Fig. 9B and C). Peak weight loss was ~−22% between 5 and 6 dpi, and all mice met the euthanasia criteria by 7 dpi (Fig. 9D and E).

Mice infected with VACV-Lis and VACV-NYCBH exhibited disease beginning at 4 dpi with peak scores of ~2.3 at 6 to 7 dpi (Fig. 9B). The decline in body temperature was observed at 3 dpi with a peak decline of ~−5°F at 5 dpi and ~−11°F at 7 dpi for the VACV-Lis and VACV-NYCBH infected groups, respectively (Fig. 9C). Both groups lost weight starting at 3 dpi, and the peak weight loss was ~−27% between 7 and 8 dpi (Fig. 9D). Ninety percent of infected mice in both groups met the euthanasia criteria between 7 to 10 dpi (Fig. 9E). In contrast to VACV infection, none of the mice in the mock and TNX-801 groups exhibited any disease, and all mice survived the 14-day study (Fig. 9B through E).

**Fig 10:**
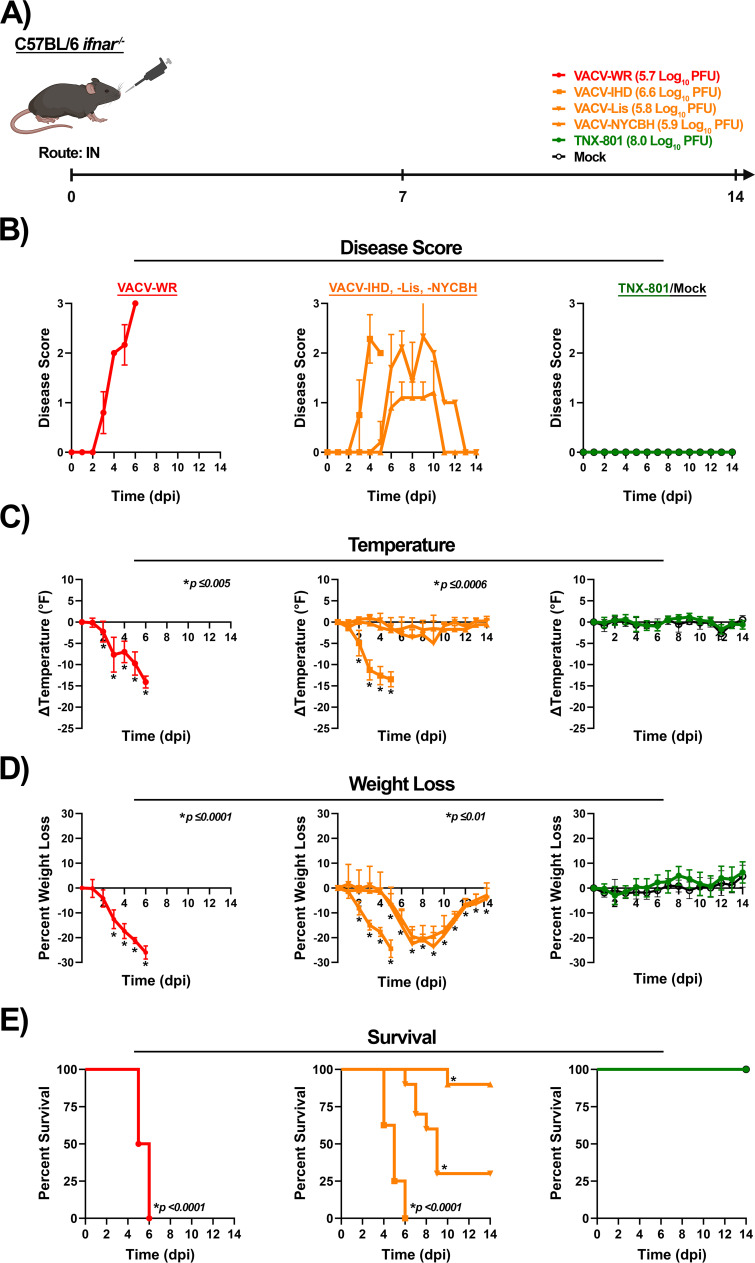
Infection of C57BL/6 *Ifnar^−/−^* mice with VACV-WR, VACV-IHD, VACV-Lis, VACV-NYCBH, TNX-801, and PBS via intranasal (IN) route (**A**). Mice were infected with VACV-WR, VACV-IHD, VACV-Lis, and VACV-NYCBH at 6.0 log_10_ PFU/mouse dose. Mice were infected with TNX-801 at 8.0 log_10_ PFU/mouse dose. Following infection, mice were monitored for disease score (**B**), body temperature (**C**), weight loss (**D**), and survival (**E**). The target dose for each virus was verified via the plaque assay, and values are provided (**A**). *p*-values comparing VACV strains to TNX-801 and mock groups are provided in each graph.

C56BL/6 *Ifnar*^−/−^ mice were next infected with VACV strains and TNX-801 at 6.0 and 8.0 log_10_ PFU/mouse, respectively (Fig. 10A). VACV-WR- and VACV-IHD-infected mice exhibited disease starting at 3 dpi with a peak disease score of ~2.3 to 3.0 between 4 and 6 dpi (Fig. 10B). Body temperature and weight loss began to decline at 2 dpi (Fig. 10C and D). Peak temperature decline was ~−14°F between 5 and 6 dpi, and peak weight loss was ~−26% between 5 and 6 dpi (Fig. 10C and D). All mice met the euthanasia criteria by 6 dpi (Fig. 10E).

Mice infected with VACV-Lis and VACV-NYCBH exhibited delayed onset of disease by 2 to 3 days (Fig. 10B). The peak disease score ranged from ~1.2 to ~2.3 between 9 and 10 dpi in VACV-Lis- and VACV-NYCBH-infected mice, respectively (Fig. 10B). Minimal alterations in temperature were observed in VACV-Lis-infected mice, whereas peak decline in temperature was ~−5°F at 9 dpi in VACV-NYCBH-infected mice (Fig. 10C). Onset of weight loss was observed at 5 dpi with peak weight loss of ~−21% to −24% between 8 and 9 dpi (Fig. 10D). Seventy percent of VACV-Lis-infected mice and 10% of VACV-NYCBH-infected mice met the euthanasia criteria by 10 dpi (Fig. 10E). In contrast to VACV-infected mice, mock and TNX-801-infected mice did not exhibit any disease (Fig. 10B through E).

**Fig 11:**
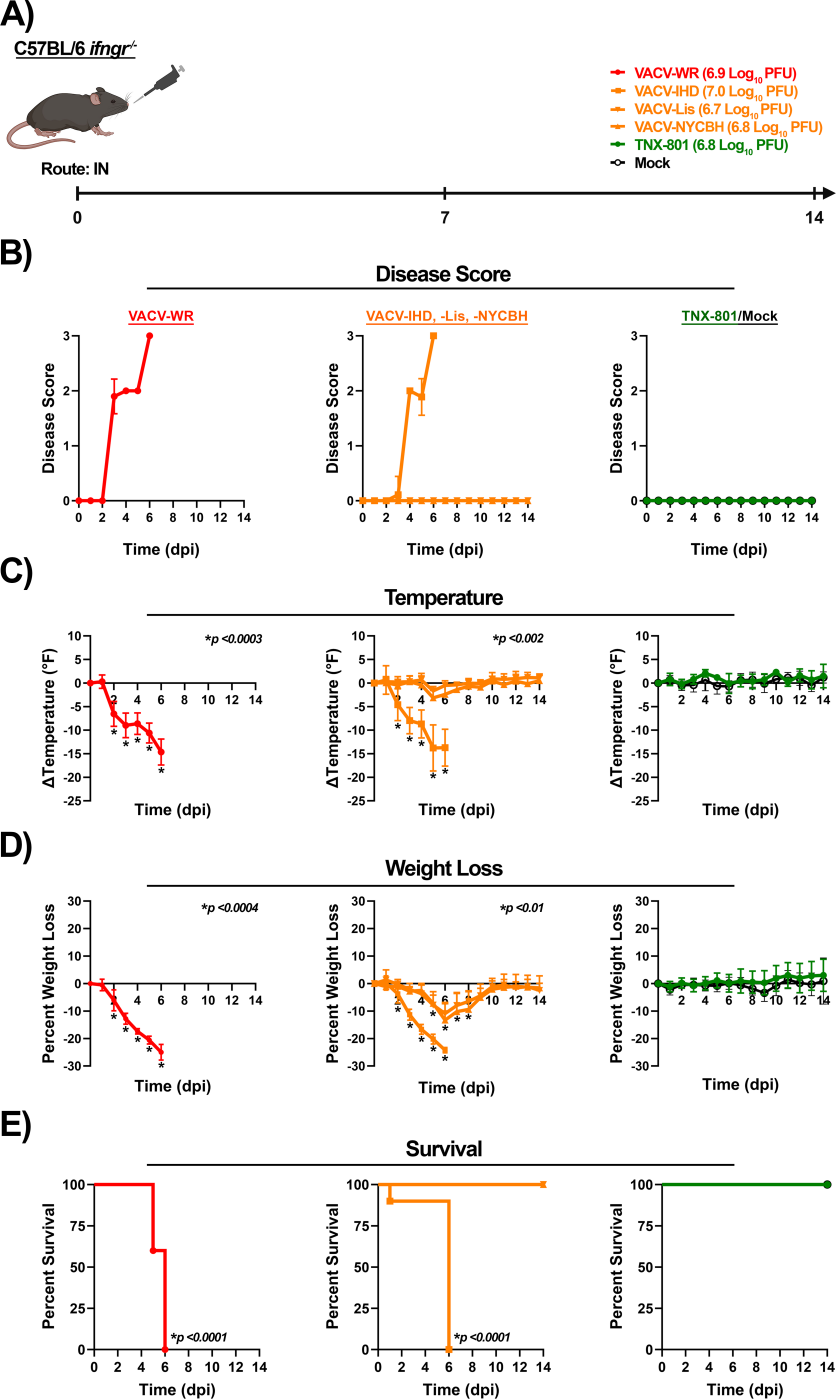
Infection of C57BL/6 *Ifngr^−/−^* mice at 7.0 log_10_ PFU/mouse dose with VACV-WR, VACV-IHD, VACV-Lis, VACV-NYCBH, TNX-801, and PBS via intranasal (IN) route (**A**). Following infection, mice were monitored for disease score (**B**), body temperature (**C**), weight loss (**D**), and survival (**E**). The target dose for each virus was verified via the plaque assay, and values are provided (**A**). *p*-values comparing VACV strains to TNX-801 and mock groups are provided in each graph.

C56BL/6 *Ifngr*^−/−^ mice were infected with VACV strains and TNX-801 at 7.0 log_10_ PFU/mouse via the intranasal route (Fig. 11A). VACV-WR- and VACV-IHD-infected mice exhibited an increase in disease score at 3 dpi with a peak disease score of ~3 at 6 dpi (Fig. 11B). Body temperature began to decline at 2 dpi with peak temperature decline of ~−15°F between 5 and 6 dpi (Fig. 11C). Weight loss was exhibited by 2 dpi with peak weight loss of ~−25% at 6 dpi, and all mice met the euthanasia criteria by 6 dpi (Fig. 11D and E). Mice infected with VACV-Lis and VACV-NYCBH exhibited mild disease (Fig. 11B). Modest decline in temperature was observed of ~−3°F at 5 dpi in both groups (Fig. 11C). Mice exhibited transient weight loss between 5 and 6 dpi with peak weight loss of ~−11% to −13%, and all mice survived the 14-day study (Fig. 11D and E). In contrast to all VACV-infected mice, the mock and TNX-801-infected mice did not exhibit any disease (Fig. 11B through E).

### Investigating infection of VACV strains and TNX-801 in C56BL/6 *Ifnar*^−/−^/*Ifngr*^−/−^ mice via the IN route

**Fig 12:**
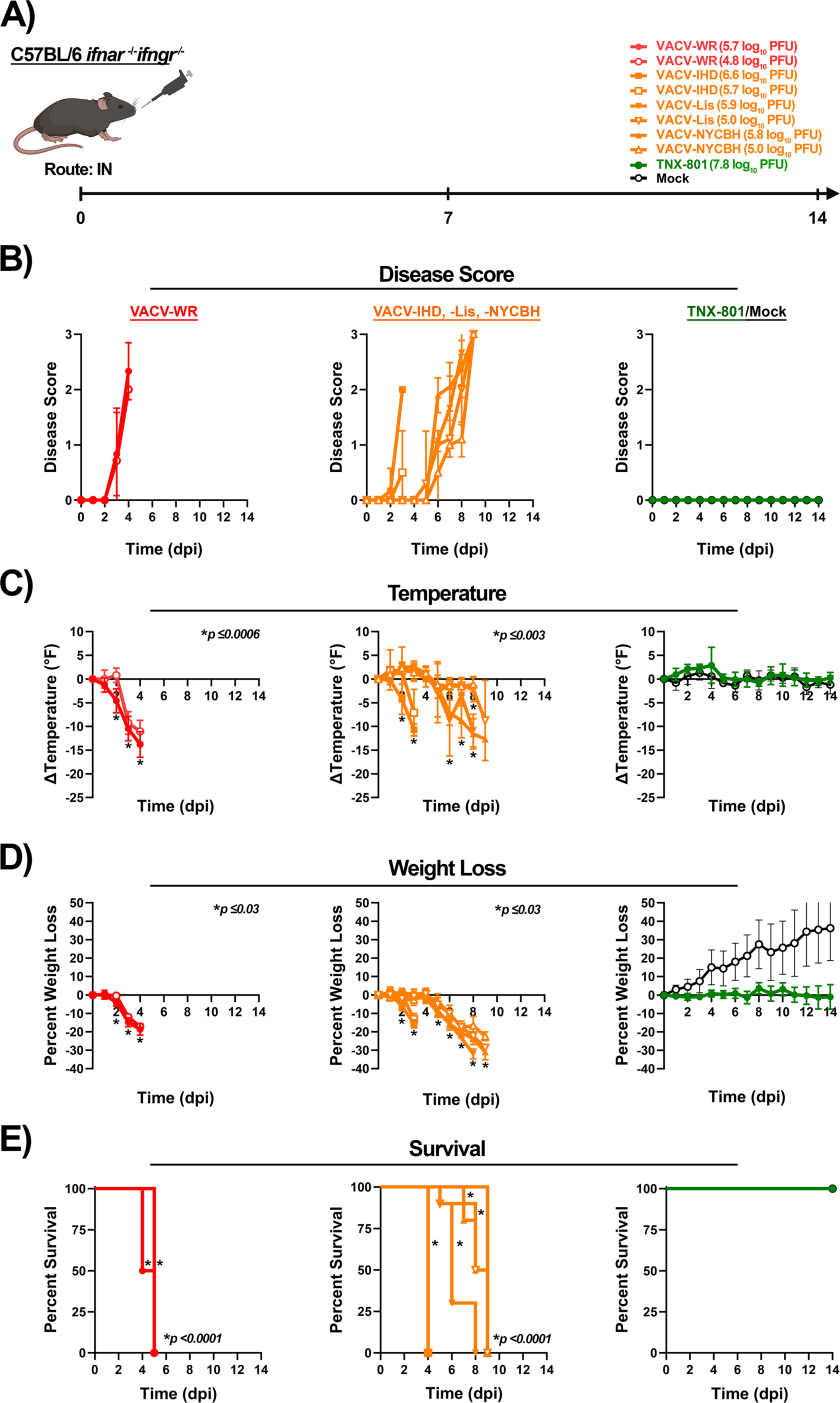
Infection of C57BL/6 *Ifnar^−/−^/Ifngr^−/−^* mice with VACV-WR, VACV-IHD, VACV-Lis, VACV-NYCBH, TNX-801, and PBS via intranasal (IN) route (**A**). Mice were infected with VACV-WR, VACV-IHD, VACV-Lis, and VACV-NYCBH at 5.0 and 6.0 log_10_ PFU/mouse dose. Mice were infected with TNX-801 at 8.0 log_10_ PFU/mouse dose. Following infection, mice were monitored for disease score (**B**), body temperature (**C**), weight loss (**D**), and survival (**E**). The target dose for each virus was verified via the plaque assay, and values are provided (**A**). *p*-values comparing VACV strains to TNX-801 and mock groups are provided in each graph.

To further investigate phenotypic differences between VACV strains and TNX-801, susceptibility to virus infection was determined in C56BL/6 *Ifnar*^−/−^/*Ifngr*^−/−^ mice via the IN route (Fig. 12A). Mice were infected at 6.0 and 5.0 log_10_ PFU/mouse with VACV strains and at 8.0 log_10_ PFU/mouse with TNX-801 (Fig. 12A).

Following VACV infection, all mice at 6.0 log_10_ PFU/mouse dose exhibited disease by 3 to 6 dpi. VACV-WR- and VACV-IHD-infected mice displayed peak scores of ~2.3 and 2.0 at 3 to 4 dpi, respectively (Fig. 12B). Body temperature began to decline between 1 and 2 dpi and then accelerated with peak decline ranging from ~−10 to −14°F between 3 and 4 dpi (Fig. 12C). Similar kinetics were observed for weight loss with peak weight loss of ~−16% to −19% between 3 and 4 dpi (Fig. 12D). All mice met the euthanasia criteria by 5 dpi (Fig. 12E). Mice infected with VACV-Lis and VACV-NYCBH exhibited delayed disease kinetics by 2 days (Fig. 12B). The onset of disease was between 5 and 6 dpi with peak scores of ~2.6 to 3 between 8 and 9 dpi (Fig. 12B). Body temperature declined at 5 dpi, and peak decline ranged from ~−11 to −13°F between 8 and 9 dpi (Fig. 12C). Similarly, weight loss began to decline at 5 dpi with peak weight loss of ~−32% between 8 and 9 dpi (Fig. 12D). All mice met the euthanasia criteria by 9 dpi (Fig. 12E).

Infection with VACV strains at 5.0 log_10_ PFU/mouse dose yielded a similar pattern. VACV-WR and VACV-IHD exhibited disease by 3 dpi (Fig. 12B). The peak disease score was ~0.5 and 2.0 at 3 to 4 dpi (Fig. 12B). Body temperature began to decline in both groups by 3 dpi with peak decline ranging from ~−7 to −11°F between 3 and 4 dpi (Fig. 12C). Mice exhibited weight loss by 3 dpi with peak weight loss of ~−13% to −17% between 3 and 4 dpi, and all mice met the euthanasia criteria by 5 dpi (Fig. 12D and E). VACV-Lis- and NYCBH-infected mice exhibited disease by 5 to 6 dpi with peak scores of ~3 at 9 dpi (Fig. 12B). Mice exhibited temperature decline by 5 dpi with peak declines of ~−1.5 to −9°F between 7 and 9 dpi (Fig. 12C). Weight loss also declined by 5 dpi with peak weight loss of ~−22% to −29% at 9 dpi (Fig. 12D). All mice met the euthanasia criteria by 9 dpi (Fig. 12E).

In contrast to VACV infection, TNX-801- and mock-infected mice did not exhibit any disease or decrease in body temperature or weight loss, and all mice survived the study (Fig. 12E).

### Investigating VACV-IHD, VACV-NYCBH, and TNX-801 replication in various tissues/organs following infection via the IN route in C56BL/6 *Ifnar*^−/−^ and C56BL/6 *Ifnar*^−/−^/*Ifngr*^−/−^ mice

**Fig 13:**
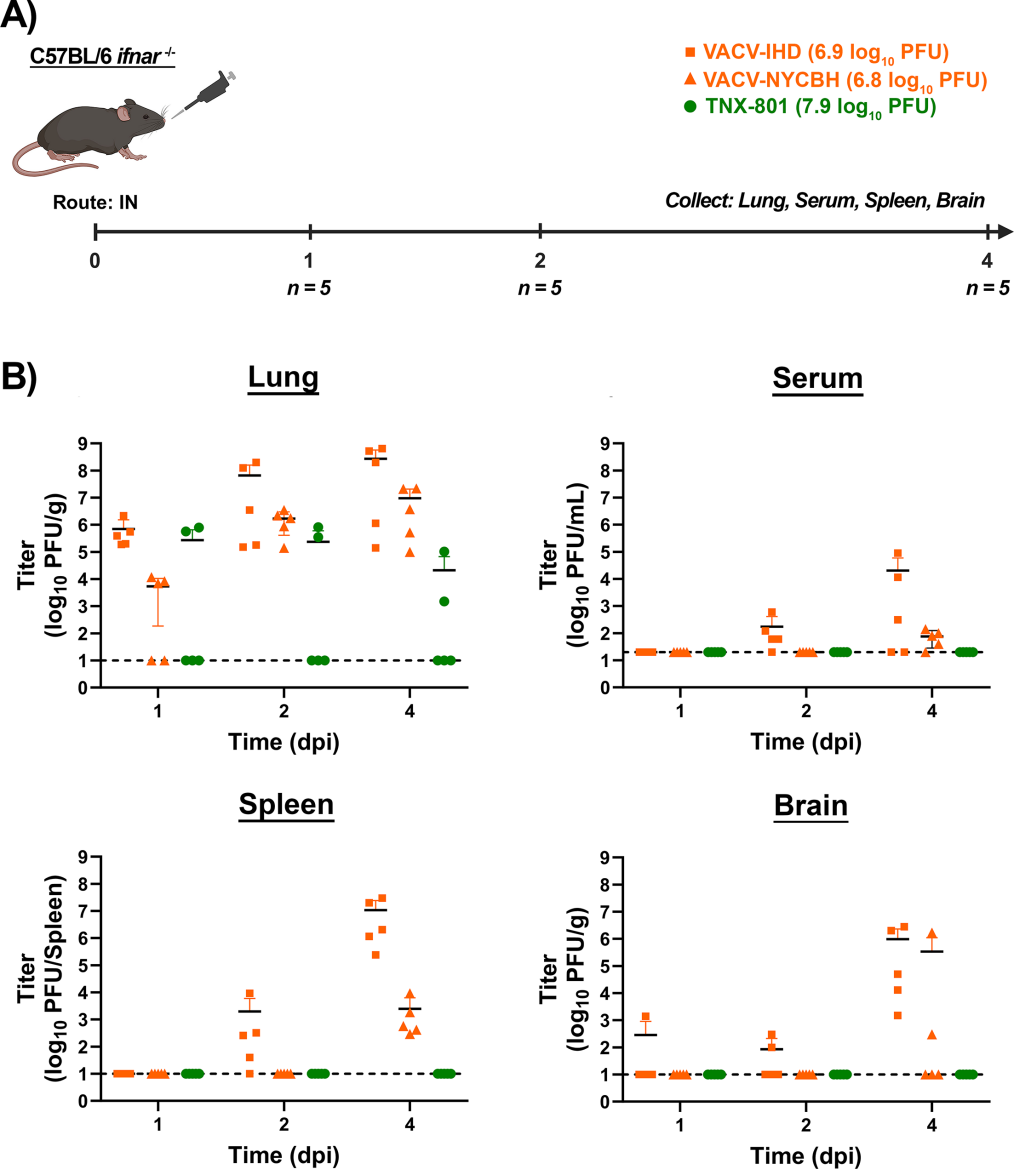
Infection of C57BL/6 *Ifnar^−/−^* mice with VACV-IHD, VACV-NYCBH, and TNX-801 via intranasal (IN) route (**A**). Mice were infected with VACV-IHD and VACV-NYCBH at 7.0 log_10_ PFU/mouse dose. Mice were also infected with TNX-801 at 8.0 log_10_ PFU/mouse dose. Following infection, groups of five mice were euthanized at 1, 2, and 4 dpi, and lung, serum, spleen, and brain tissues were harvested (**A**). All samples were subjected to three cycles of freeze/thaw/sonication and titrated on BSC-40 cells. The target dose for each virus was verified via the plaque assay, and values are provided (**A**). The limit of detection is shown for the infectious assay (1.0 log_10_ PFU/g, 1.0 log_10_ PFU/spleen, or 1.3 log_10_ PFU/mL) with black dashed lines.

C56BL/6 *Ifnar*^−/−^ mice were infected with 8.0 (TNX-801) and 7.0 log_10_ PFU (VACV-IHD and NYCBH) via the IN route. Lungs, serum, spleen, and brain were collected at 1, 2, and 4 dpi (Fig. 13A). VACV-IHD and -NYCBH replicated rapidly within the lungs. Average VACV-IHD titers were ~6.0 at 1 dpi, ~8.0 at 2 dpi, and ~8.4 log_10_ PFU/g at 4 dpi (Fig. 13B). The replication of VACV-NYCBH in the lung displayed delayed replication kinetics relative to VACV-IHD. Average VACV-NYCBH titers were ~3.8 at 1 dpi, ~6.3 at 2 dpi, and ~7.2 log_10_ PFU/g at 4 dpi (Fig. 13B).

Similar to the lung, VACV-IHD displayed faster replication kinetics relative to VACV-NYCBH in serum, spleen, and brain. At 1 dpi, no infectious virus was detected in serum or spleen of VACV-IHD-infected mice. At 2 and 4 dpi, average VACV-IHD titers in serum ranged from ~2.3 and ~4.2 log_10_ PFU/mL, respectively (Fig. 13B). Average VACV-IHD titers in the spleen ranged from ~3.4 and ~7.2 log_10_ PFU/spleen at 2 and 4 dpi, respectively (Fig. 13B). VACV-IHD was detected in the brain at all three time-points with average VACV-IHD titers of ~2.5 at 1 dpi, ~1.8 at 2 dpi, and ~6.1 log_10_ PFU/g at 4 dpi (Fig. 13B). The serum, spleen, and brain samples from VACV-NYCBH-infected mice were below the limit of detection at 1 and 2 dpi (Fig. 13B). Average VACV-NYCBH titers in the serum, spleen, and brain ranged from ~1.8 to ~5.5 log_10_ PFU/mL or /spleen or /g at 4 dpi (Fig. 13B).

In contrast to VACV-IHD and -NYCBH, TNX-801 was only detected in the lung of infected mice with average TNX-801 titers of ~5.5 at 1 dpi, ~5.3 at 2 dpi, and ~4.3 log_10_ PFU/g at 4 dpi (Fig. 13B).

**Fig 14:**
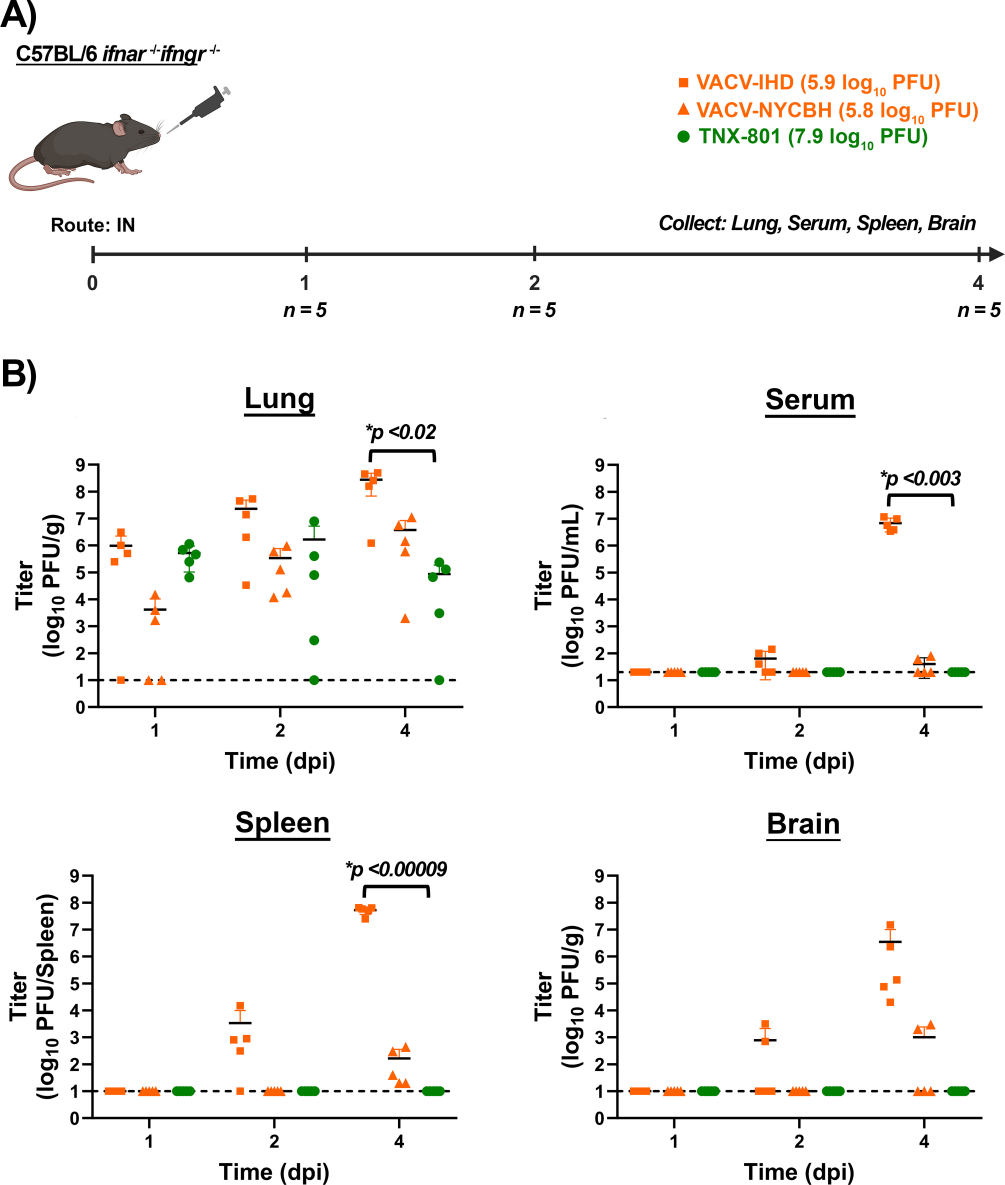
Infection of C57BL/6 *Ifnar^−/−^/Ifngr^−/−^* mice with VACV-IHD, VACV-NYCBH, and TNX-801 via intranasal (IN) route (**A**). Mice were infected with VACV-IHD and VACV-NYCBH at 7.0 log_10_ PFU/mouse dose. Mice were also infected with TNX-801 at 8.0 log_10_ PFU/mouse dose. Following infection, groups of five mice were euthanized at 1, 2, and 4 dpi, and lung, serum, spleen, and brain tissues were harvested (**A**). All samples were subjected to three cycles of freeze/thaw/sonication and titrated on BSC-40 cells. The target dose for each virus was verified via the plaque assay, and values are provided (**A**). The limit of detection is shown for the infectious assay (1.0 log_10_ PFU/g, 1.0 log_10_ PFU/spleen, or 1.3 log_10_ PFU/mL) with black dashed lines. *p*-values comparing TNX-801 and VACV-IHD are provided in graphs.

C56BL/6 *Ifnar*^−/−^/*Ifngr*^−/−^ mice were infected with 8.0 (TNX-801) and 6.0 and log_10_ PFU (VACV-IHD and NYCBH) via the IN route. Similar to the C56BL/6 *Ifnar*^−/−^ study, lungs, serum, spleen, and brain were collected at 1, 2, and 4 dpi (Fig. 14A). Average VACV-IHD titers in the lungs were ~6.0 at 1 dpi, ~7.4 at 2 dpi, and ~8.5 log_10_ PFU/g at 4 dpi (Fig. 14B). The replication of VACV-NYCBH in the lung displayed delayed replication kinetics relative to VACV-IHD. Average VACV-NYCBH titers were ~3.6 at 1 dpi, ~5.5 at 2 dpi, and ~6.6 log_10_ PFU/g at 4 dpi (Fig. 14B).

Similar to the C56BL/6 *Ifnar*^−/−^ study, VACV-IHD displayed faster replication kinetics relative to VACV-NYCBH in serum, spleen, and brain. At 1 dpi, no infectious virus was detected in serum, spleen, and brain of VACV-IHD-infected mice. Average VACV-IHD titer in serum ranged from ~1.8 and 6.8 log_10_ PFU/mL at 2 and 4 dpi, respectively (Fig. 14B). Average VACV-IHD titer in the spleen ranged from ~3.5 and 7.8 log_10_ PFU/spleen at 2 and 4 dpi, respectively (Fig. 14B). Average VACV-IHD titer in the brain ranged from ~2.8 and 6.4 log_10_ PFU/g at 2 and 4 dpi, respectively (Fig. 14B). The serum, spleen, and brain samples from VACV-NYCBH-infected mice were below the limit of detection at 1 and 2 dpi (Fig. 14B). Average VACV-NYCBH titers in the serum, spleen, and brain ranged from ~1.6 to 3.0 log_10_ PFU/mL or /spleen or /g at 4 dpi (Fig. 14B).

In contrast to VACV-IHD and -NYCBH, TNX-801 was only detected in the lungs of infected mice with average TNX-801 titers of ~5.7 at 1 dpi, ~6.1 at 2 dpi, and ~4.8 log_10_ PFU/g at 4 dpi (Fig. 14B).

## DISCUSSION

Comprehensive studies comparing the phenotypic difference between VACV strains and TNX-801 or with other Orthopoxviruses are either lacking or limited; consequently, few comparisons can be made (36). In this study, we compared the plaque phenotype of TNX-801 with multiple VACV strains as well as MVA. TNX-801 consistently yielded a small plaque phenotype in NHP cell lines than VACV strains. In addition, the plaque size of VACV strains increased in size through 4 dpi yielding a large plaque phenotype on BSC-40 cells, whereas the plaque size of TNX-801 did not increase between 3 and 4 dpi. In contrast, the plaque size of all viruses continued to increase through 6 dpi on Vero-E6 cells. In addition, the plaque phenotype was more discernable between 4 and 6 dpi for all viruses, particularly for TNX-801 and MVA, suggesting that Vero-E6 cells may be a superior cell substrate to visualize plaques.

Data regarding VACV infection in human primary cells are limited (37–39). This study investigated the infection of multiple VACV strains as well as MVA in eight human primary cell lines from two main routes of poxvirus transmission, dermal and respiratory tracts. All eight cell lines were susceptible to infection with VACV strains. VACV rapidly replicated its genome within 12 hpi, and infectious progeny was produced within 24 hpi with peak virus titers by 48 to 96 hpi. Lastly, similar to previous studies, MVA displayed limited genome replication and did not produce infectious progeny in human primary cells (40–42).

TNX-801 was also able to productively infect all eight human primary cells. The replication kinetics were comparable to VACV strains; however, the magnitude of infectious virus was ~10- to 100-fold lower in the majority of the cell lines. These data suggest that TNX-801 is ~10- to 100-fold more attenuated in both dermal and respiratory tract cells that would serve as the initial site of replication during virus infection and, thus, would limit TNX-801 replication and subsequent dissemination. The lack of replication and disseminated disease following intranasal TNX-801 infection in murine models supports this hypothesis.

The comparable fitness of TNX-801 and VACV strains in primary human skeletal muscle cells has important implications regarding the optimal route of vaccination. The intramuscular vaccination of TNX-801 may provide comparable immunogenicity to VACV strains used in smallpox eradication. TNX-801 vaccination studies in rabbits and NHPs have focused on the scarification route to yield robust humoral responses as well as efficacy against MPXV and SARS-CoV-2 challenges (36, 43, 44). Intramuscular TNX-801 vaccination may provide superior humoral responses relative to other routes and warrants further investigation.

The *in vitro* studies provided insights into the potential mechanism/s of TNX-801 attenuation during the virus life cycle. The virus life cycle comprises of three main stages: attachment/entry, genome replication, and assembly/egress. The kinetics of genome replication in immortalized NHP and human primary cell lines was comparable between TNX-801 and VACV strains suggesting that the potential mechanism of attenuation is not at the attachment/entry and genome replication stages. However, the infectious particle-to-genome copy ratio data suggests that TNX-801 genome packaging is ~10- to 100-fold less efficient than the VACV strains. These data strongly indicate that the potential mechanism of TNX-801 attenuation is at the assembly/egress stage, and studies are underway to further dissect the mechanism.

Wild-type and immunocompromised murine models including severe combined immunodeficiency disease (SCID) mice have been previously investigated for VACV infection (45–64). However, the type I and II interferon receptor knockout mice have not been previously investigated. Interferons alpha and gamma play a critical role in the host response against viral pathogens, and the disruption of either or both pathways dramatically increases the susceptibility of mice to virus infection (65–72). The data from this study demonstrate that immunocompromised mouse models consisting of interferon alpha and/or gamma receptor knockout were susceptible to VACV infection. However, the severity of disease was dependent on the route of infection. Infection in C56BL/6 *Ifnar*^−/−^ and C56BL/6 *Ifngr*^−/−^ via the intraperitoneal route did not produce uniform disease. VACV-WR and VACV-IHD were able to produce severe disease including lethality, whereas minimal/no disease was observed with VACV-Lis and VACV-NYCBH infection. In contrast, mice exhibited mild to severe disease following intranasal infection with VACV strains in both murine models. The C56BL/6 *Ifnar*^−/−^ model was more sensitive via intranasal VACV infection than C56BL/6 *Ifngr*^−/−^. VACV infection at 7.0 and 6.0 log_10_ PFU doses induced lethal disease in the C56BL/6 *Ifnar*^−/−^ model regardless of strain, whereas the VACV infection at 7.0 log_10_ PFU dose in the C56BL/6 *Ifngr*^−/−^ model only produced lethal disease with VACV-WR and VACV-IHD. Mice infected with VACV-Lis and VACV-NYCBH exhibited mild disease with alterations in body temperature and weight. Not surprisingly, the C56BL/6 *Ifnar*^−/−^/*Ifngr*^−/−^ model was considerably more sensitive to VACV infection than either C56BL/6 *Ifnar*^−/−^ or C56BL/6 *Ifngr*^−/−^ models. All mice exhibited severe disease and met euthanasia criteria regardless of VACV strain or dose. These data suggest that the C56BL/6 *Ifnar*^−/−^/*Ifngr*^−/−^ model is at least 10-fold more sensitive to VACV infection than either C56BL/6 *Ifnar*^−/−^ or C56BL/6 *Ifngr*^−/−^ models. Taken together, these data demonstrate that C56BL/6 *Ifnar*^−/−^, C56BL/6 *Ifngr*^−/−^, and C56BL/6 *Ifnar*^−/−^/*Ifngr*^−/−^ are susceptible to VACV infection via IP or IN routes and can produce severe disease including lethality.

All three murine models were able to differentiate phenotypic differences between VACV strains. VACV-WR and VACV-IHD strains were the most virulent as they were able to rapidly cause lethal disease in all mouse strains regardless of the dose or route, whereas VACV-Lis- and VACV-NYCBH-infected mice exhibited either minimal/mild disease or delayed kinetics of disease and lethality. These data demonstrate that VACV-WR and VACV-IHD strains are at least 10-fold more virulent than VACV-Lis and VACV NYCBH. In addition, only 10% of the mice infected with VACV-NYCBH at 6.0 log_10_ PFU in the C56BL/6 *Ifnar*^−/−^ mouse model met the euthanasia criteria indicating that VACV-NYCBH is more attenuated than VACV-WR, VACV-IHD, and VACV-Lis.

In contrast to VACV strains, TNX-801 was unable to cause any disease in all three murine models, and all parameters measured were similar to mock-treated groups. The phenotypic difference was more pronounced in the C56BL/6 *Ifnar*^−/−^/*Ifngr*^−/−^ model where uniform lethality was achieved regardless of the dose with all VACV strains. These data demonstrate that TNX-801 is >10- to 1,000-fold more attenuated than VACV-WR, VACV-IHD, VACV-Lis, and VACV-NYCBH. Further studies are underway to determine the lethal dose (LD_50_) of all VACV strains in both C56BL/6 *Ifnar*^−/−^ and C56BL/6 *Ifnar*^−/−^/*Ifngr*^−/−^ models to ascertain the precise level of TNX-801 attenuation.

The magnitude of TNX-801, VACV-IHD, and VACV-NYCBH replication was investigated in C56BL/6 *Ifnar*^−/−^ and C56BL/6 *Ifnar*^−/−^/*Ifngr*^−/−^ mice. Mice infected with TNX-801 at 10-fold (C56BL/6 *Ifnar*^−/−^) or 100-fold (C56BL/6 *Ifnar*^−/−^/*Ifngr*^−/−^) higher doses than VACV strains produced limited replication in the lung and did not produce disseminated systemic infection. In contrast, both VACV-IHD and VACV-NYCBH rapidly replicated in the lungs and disseminated to other organs and tissues causing systemic infection. The infectious titers of VACV-IHD and VACV-NYCBH were 4- to 1,000,000-fold higher than the limit of detection in spleen, serum, and brain tissues. These data further highlight the attenuation of TNX-801 relative to the VACV strains.

The ~90-kb central core of poxvirus genome encodes genes responsible for virus replication and is consequently highly conserved (1, 3, 4). However, the left and right arms of the genome are more variable as they encode for host range restriction and immune antagonism genes. TNX-801 genome is ~20 kb larger than VACV genome, and yet, it displays an attenuated phenotype relative to VACV (1, 3, 4, 34). The gene/s that underlie this phenotype at various stages of the poxvirus replication cycle are under investigation.

Edward Jenner originally thought the agent responsible for the mild disease in milkmaids originated in horses (36, 73–78). In addition, HPXV may have been inadvertently used as a smallpox vaccine due to animal passaging during vaccine manufacturing in the 19th and early 20th centuries (36, 73–78). Recent genomic studies of American Civil War Era smallpox vaccine scab kits showed the presence of both VACV and HPXV, with one of the isolates displaying 99.7% nucleotide identity to HPXV (74). These data suggest that HPXV was inadvertently used as a smallpox vaccine and support Jenner’s original hypothesis.

In conclusion, TNX-801 vaccine is a preclinical stage vaccine against monkeypox and smallpox infection. One critical hurdle in the development of replication-competent vaccines is the demonstration of vaccine safety in relevant *in vitro* and *in vivo* models. In this study, we investigated the potential attenuation of TNX-801 relative to VACV-based smallpox vaccine strains used in eradication. In immortalized NHP cell lines, TNX-801 displayed a small plaque phenotype, delayed replication kinetics, and >10- to 100-fold lower infectious titers. Human primary cells from dermal and respiratory tracts also showed similar levels of TNX-801 attenuation. Lastly, the TNX-801 infection of immunocompromised mice was unable to cause any disease or alteration in body temperature and weight, and all mice survived the study period. These data demonstrate that TNX-801 is >10- to 1,000-fold more attenuated than older Vaccinia virus-based vaccine strains used in smallpox eradication.

## MATERIALS AND METHODS

### Cells

Vero-E6 (CRL-1586) and BSC-40 (CRL-2761) cells were obtained from the American Type Culture Collection (ATCC). Cells were cultured in Dulbecco’s modified Eagle medium (DMEM) supplemented with 10% fetal bovine serum (FBS) and gentamicin (50 µg/mL) at 37°C and 5% CO_2_.

Normal adult human primary cells were obtained from ATCC. Primary dermal tract cells comprised of epidermal melanocytes (PCS-200-013), epidermal keratinocytes (PCS-200-011), dermal fibroblasts (PCS-201-012), and skeletal muscle (PCS-950-010). Primary respiratory tract cells were bronchial/tracheal epithelial (PCS-300-010), small airway epithelial (PCS-301-010), lung fibroblasts (PCS-201-013), and bronchial/tracheal smooth muscle (PCS-130-011). Cells were cultured and seeded in their respective basal mediums and growth kit supplements per manufacturer recommendations.

### Viruses and virus amplification

VACV strains [Western Reserve (WR), International Health Department (IHD), Lister (Lis), New York City (NYCBH), and Modified Vaccinia Ankara (MVA)] were obtained from ATCC and BEI Resources. HPXV (TNX-801) stock was obtained as previously described (36). Virus stocks were generated in BSC-40 cells in T-150 flasks seeded overnight to achieve 70% confluence. Monolayers were infected at an MOI of 0.01 and incubated for 1 hr at 37°C and 5% CO_2_. After incubation, 20 mL of growth media was added. Monolayers were harvested via cell scraping at 72 hpi and subjected to three cycles of freeze/thaw/sonication (F/T/S). For murine studies, virus stocks were sucrose-purified as previously described (35). All virus stocks were titrated by the plaque assay on BSC-40 cells.

### Virus titration

The protocol has been described previously. Plaque assays were performed on BSC-40 cell monolayers and were overlaid with 2 mL of 0.5% methylcellulose in 1× MEM, GlutaMAX supplemented with 5% FBS, and gentamicin (50 µg/mL). For plaque phenotype comparison, representative six-well plates are shown in the figures.

### Multi-step growth kinetics

BSC-40, Vero-E6, and human primary cells were seeded to achieve 50% confluence monolayers overnight in 6-well plates. Monolayers were infected at an MOI of 0.01 with VACV strains, TNX-801, and MVA. Following 1-hr incubation, monolayers were rinsed with 10 mL 1× PBS, and 2 mL of growth media was added. Monolayers from triplicate wells were harvested via cell scraping at 0, 12 (BSC-40 and Vero-E6 cells only), 24, 48, 72, and 96 hpi (except BSC-40 cells) in triplicate. Samples were subjected to three cycles of F/T/S and titrated on BSC-40 cells.

### Mouse studies

Breeding pairs of C57BL/6 *Ifnar^−/−^*, C57BL/6 *Ifngr^−/−^*, and C57BL/6 *Ifnar^−/−^*/*Ifngr^−/−^* were purchased from Jackson Laboratories to establish mouse colonies at Tonix Pharmaceuticals. Fourteen-day studies were performed to investigate the virulence of VACV strains and TNX-801. All virus stocks used in studies were sucrose-purified. Mouse-adapted (VACV-WR and VACV-IHD) as well as older VACV strains utilized in smallpox eradication (VACV-Lis and VACV-NYCBH) were utilized as positive controls. Mock-treated mice were administered 1× PBS and were used as negative control. Cohorts of 10 (5 male and five female) 4- to 6-week-old mice were infected via the IP or IN route. Viruses were diluted in 1× PBS to obtain various concentrations. Volumes to infect mice with viruses or 1× PBS were as follows: 100 µL (IP) or 30 µL (IN, 15 µL/nare) of virus or 1× PBS. C57BL/6 *Ifnar^−/−^* and C57BL/6 *Ifngr^−/−^* mice were infected with all viruses at 6.0 log_10_ PFU/mouse via the IP route. C57BL/6 *Ifnar^−/−^* mice were infected with VACV strains (7.0 and 6.0 log_10_ PFU/mouse) and TNX-801 (8.0 and 7.0 log_10_ PFU/mouse) via the IN route. C57BL/6 *Ifngr^−/−^* mice were infected with all viruses at 7.0 log_10_ PFU/mouse via the IN route. C57BL/6 *Ifnar^−/−^*/*Ifngr^−/−^* mice were infected with VACV strains at 6.0 and 5.0 log_10_ PFU/mouse and TNX-801 at 8.0 log_10_ PFU/mouse via the IN route. Following infection, mice were monitored daily for disease score, body temperature, weight loss, and survival. The disease score criteria are as follows: 0 = normal; 1 = rough coat and/or hunched posture, 20% body weight loss from baseline; 2 = mild lethargy and/or mild dyspnea, 25% body weight loss from baseline; and 3 = moderate lethargy and/or moderate dyspnea, 30% body weight loss from baseline. All target doses were verified via the plaque assay on BSC-40 cells, and verified doses are shown in respective figures.

To investigate virus replication, C56BL/6 *Ifnar*^−/−^ mice were infected with 8.0 (TNX-801) and 7.0 log_10_ PFU (VACV-IHD and NYCBH) via the IN route. C56BL/6 *Ifnar^−/−^*/*Ifngr^−/−^* mice were also infected with 8.0 (TNX-801) and 6.0 log_10_ PFU (VACV-IHD and NYCBH) via the IN route. Lungs, serum, spleen, and brain were collected at 1, 2, and 4 dpi.

Ten percent of homogenates were generated in 1× PBS and subjected to three cycles of F/T/S. All samples were titrated on BSC-40 cells. All target doses were verified via the plaque assay on BSC-40 cells, and verified doses are shown in respective figures.

All study design illustrations were generated in BioRender software.

### Statistical analysis

Statistical significance in temperature, weight loss, and survival was evaluated using nonparametric one-way analysis of variance with Tukey’s multiple comparison tests, nonparametric Mann–Whitney *t*-test, or unpaired multiple *t*-tests using a false discovery rate approach. A comparison of survival curves was performed using the log-rank (Mantel–Cox) test, the log-rank test for trend, and Gehan–Breslow–Wilcoxon tests. The representative *P-*values for each analysis are provided in the figures. All the tests were performed using GraphPad PRISM software (version 10.0.1). Statistically significant *P*-values <0.05 are shown in the graphs.

## SUPPLEMENTAL MATERIAL

Supplemental material msphere.00265-24-s0001.pdf Fig. S1 to S10.

ASM does not own the copyrights to Supplemental Material that may be linked to, or accessed through, an article. The authors have granted ASM a non-exclusive, world-wide license to publish the Supplemental Material files. Please contact the corresponding author directly for reuse.
