# Supplementary material for: Recombinant chimeric horsepox virus (TNX-801) is attenuated relative to vaccinia virus strains in both in vitro and in vivo models
Source: mSphere. 2024 Nov 13;9(12):e00265-24. doi: 10.1128/msphere.00265-24 (PMC11656774; doi:10.1128/msphere.00265-24)
Supplement: Supplemental material — Fig. S1 to S10. [file msphere.00265-24-s0001.pdf]

Supp. Figure 1

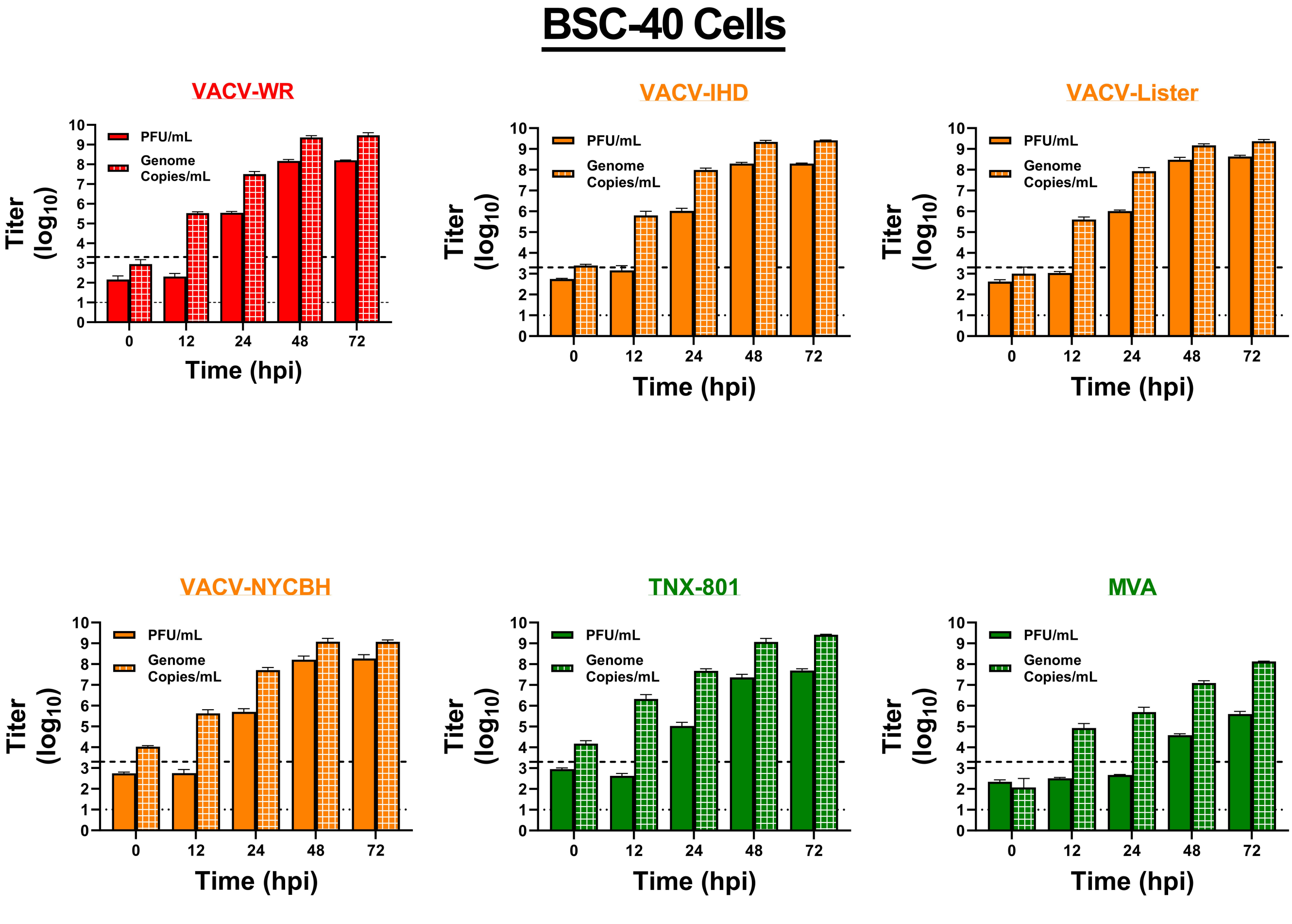

Supp. Figure 2

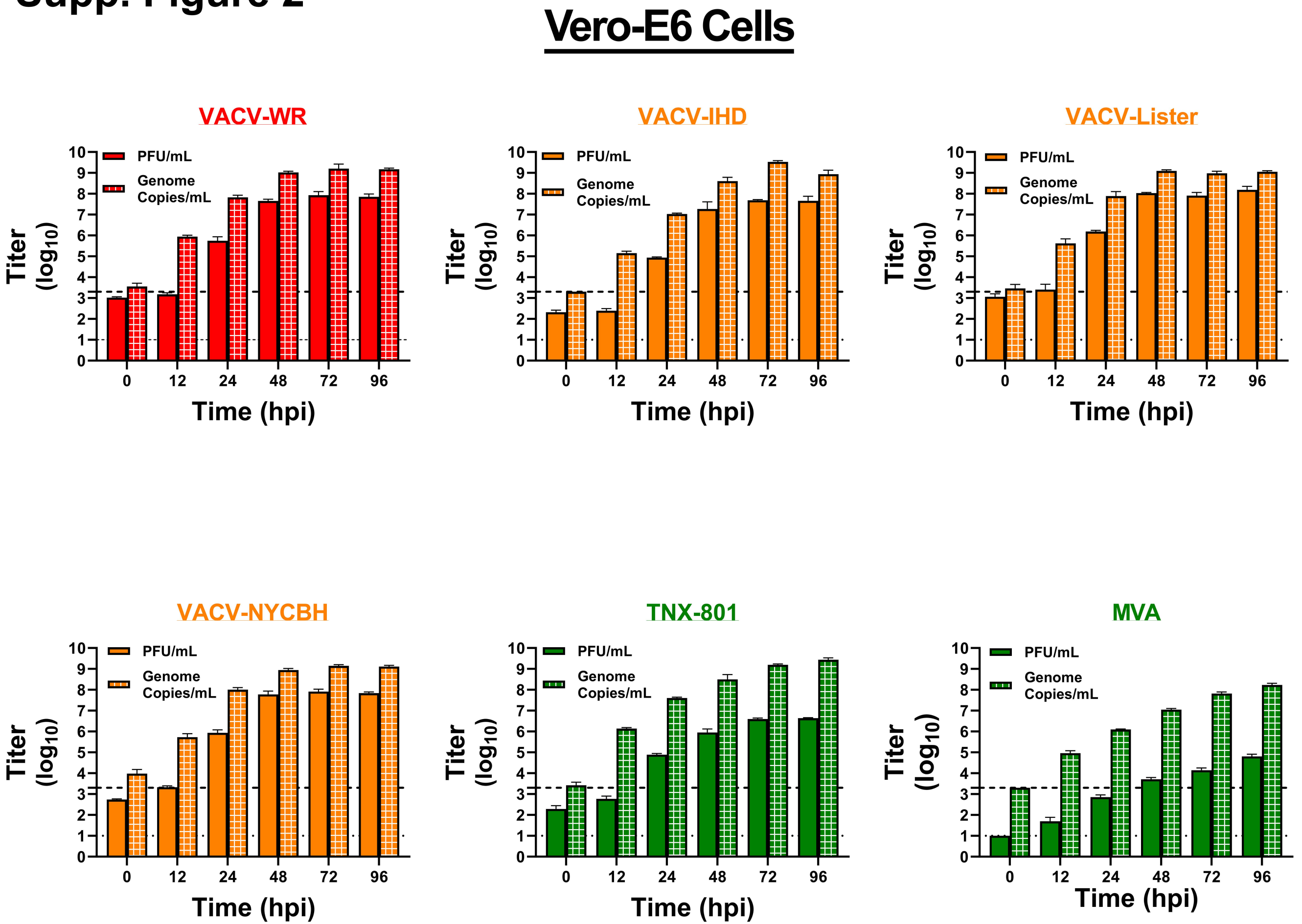

Supp. Figure 3

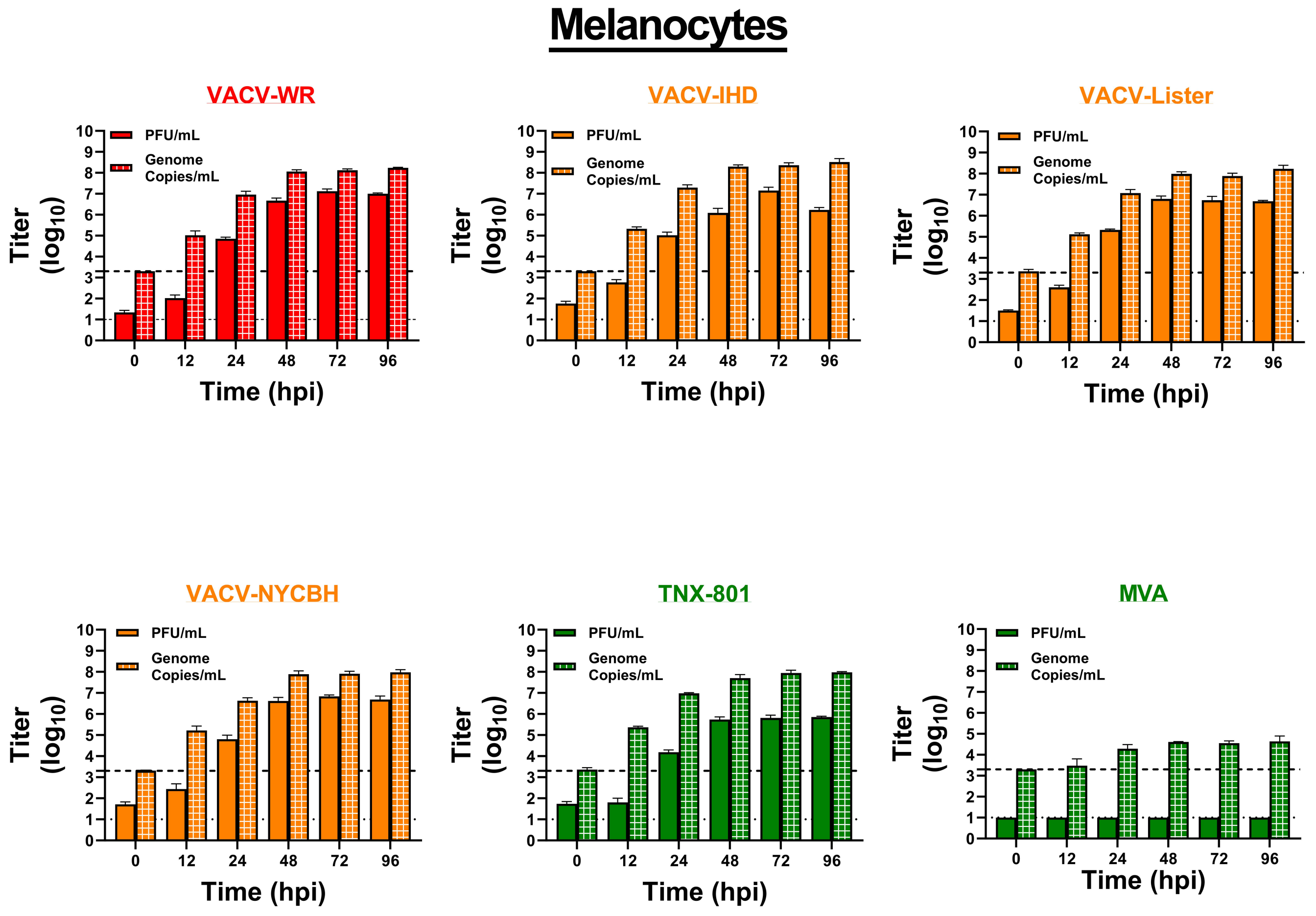

Supp. Figure 4

Keratinocytes

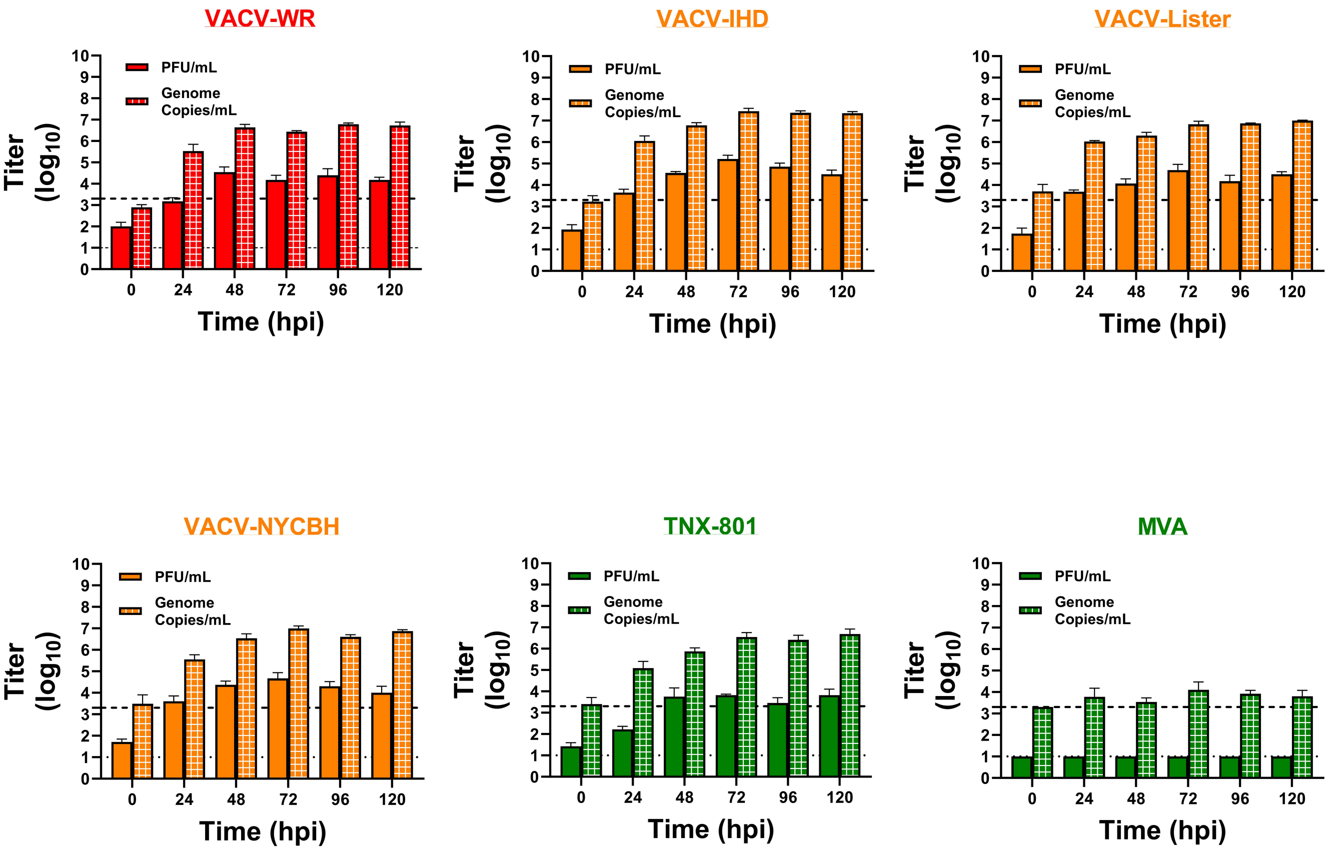

Supp. Figure 5

Dermal Fibroblasts

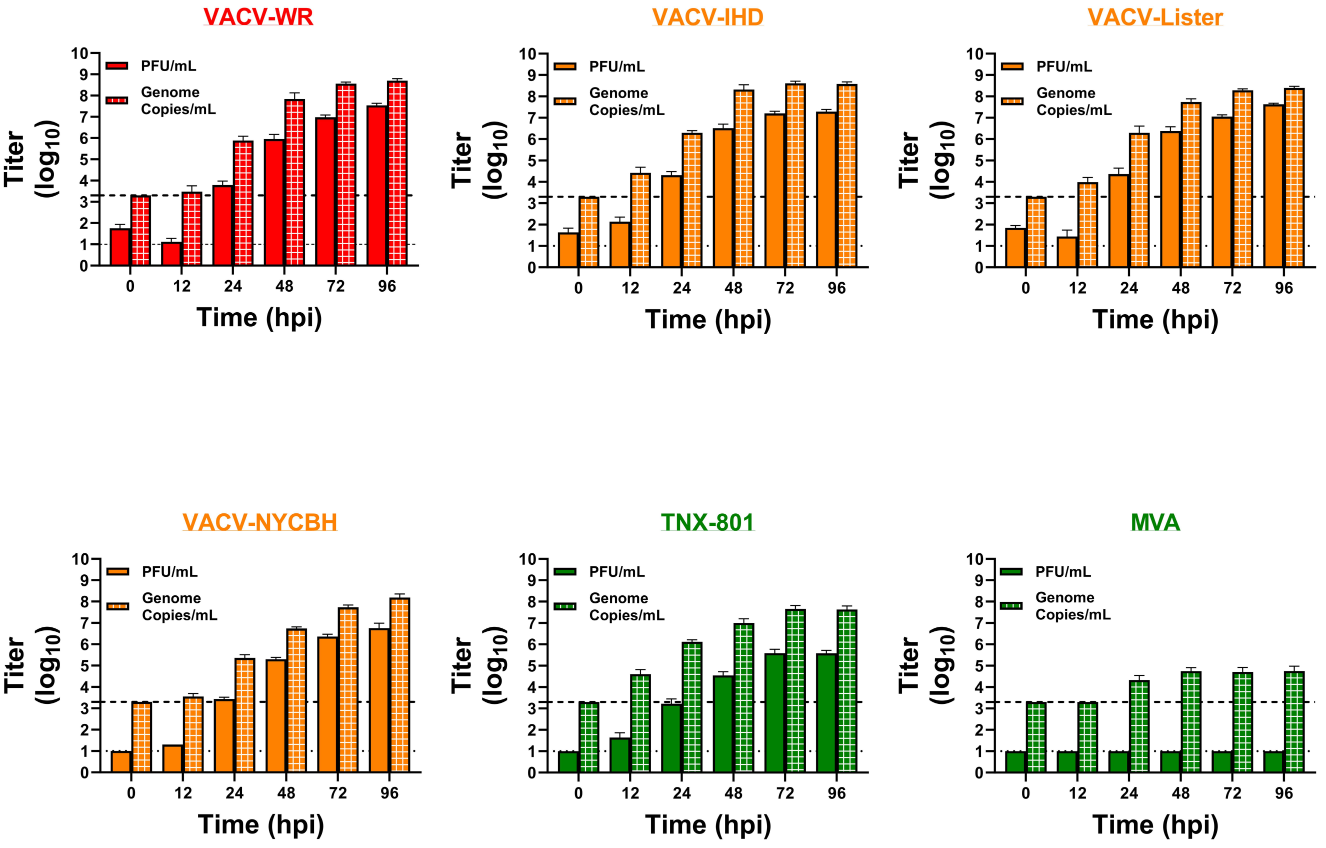

Supp. Figure 6

Skeletal Muscle Cells

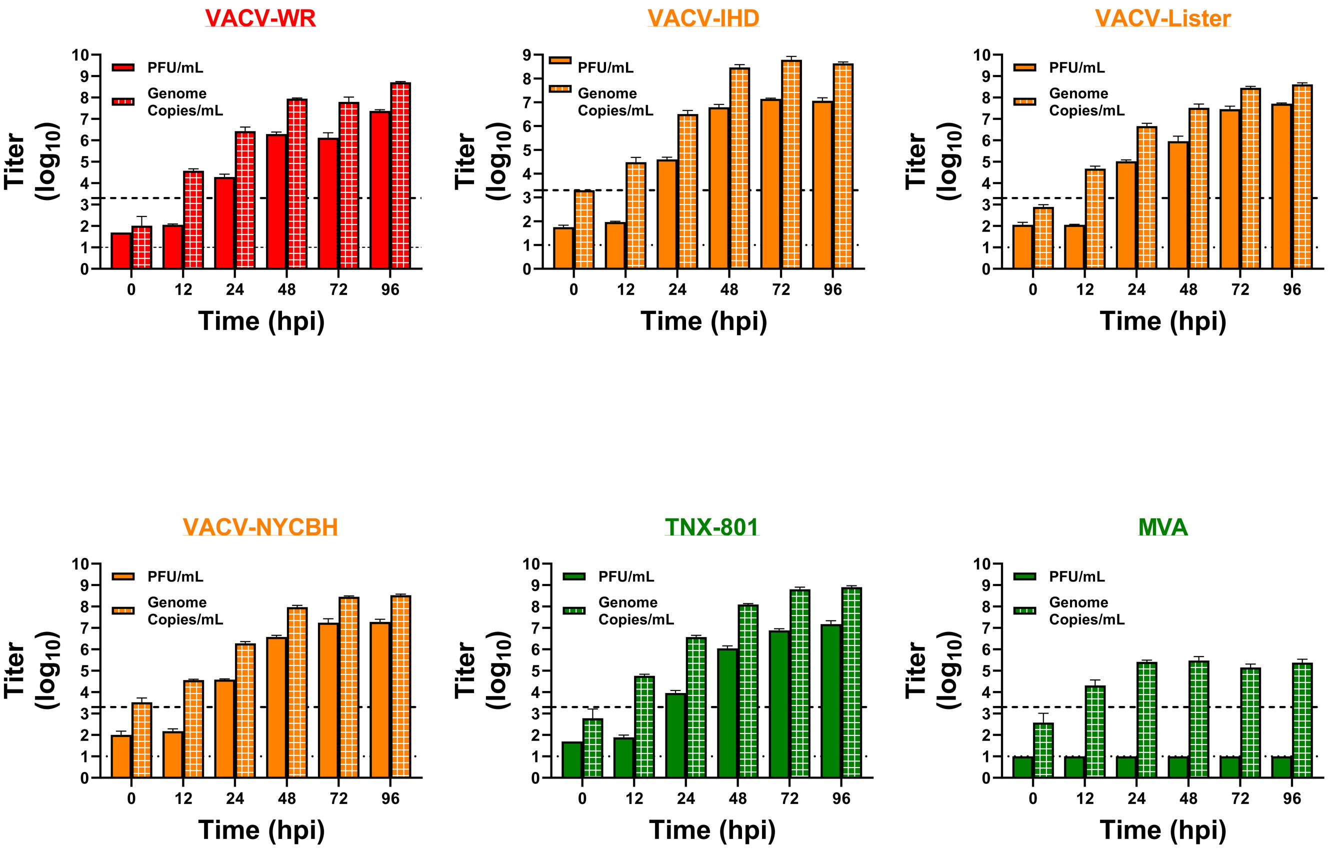

Supp. Figure 7

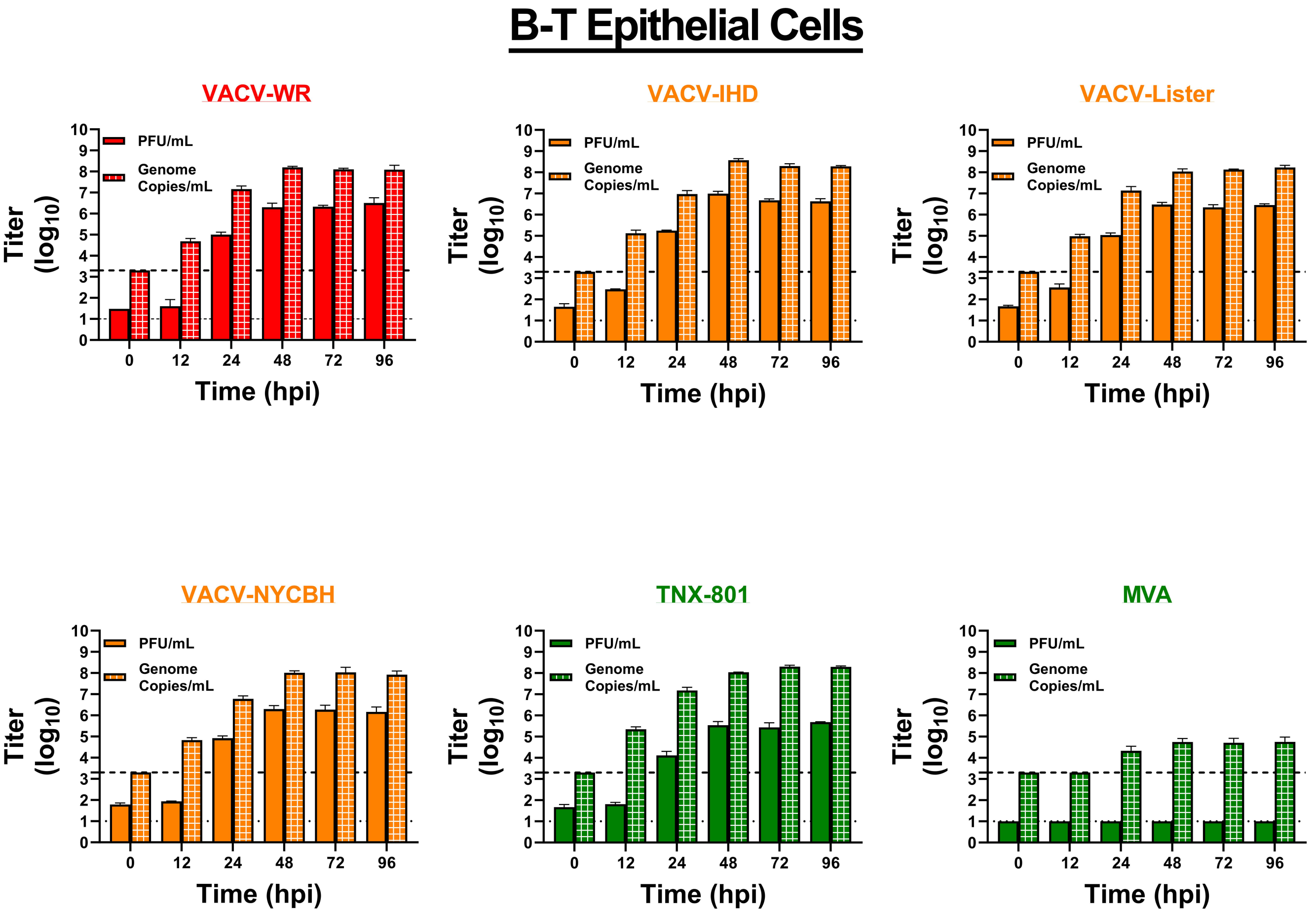

Supp. Figure 8

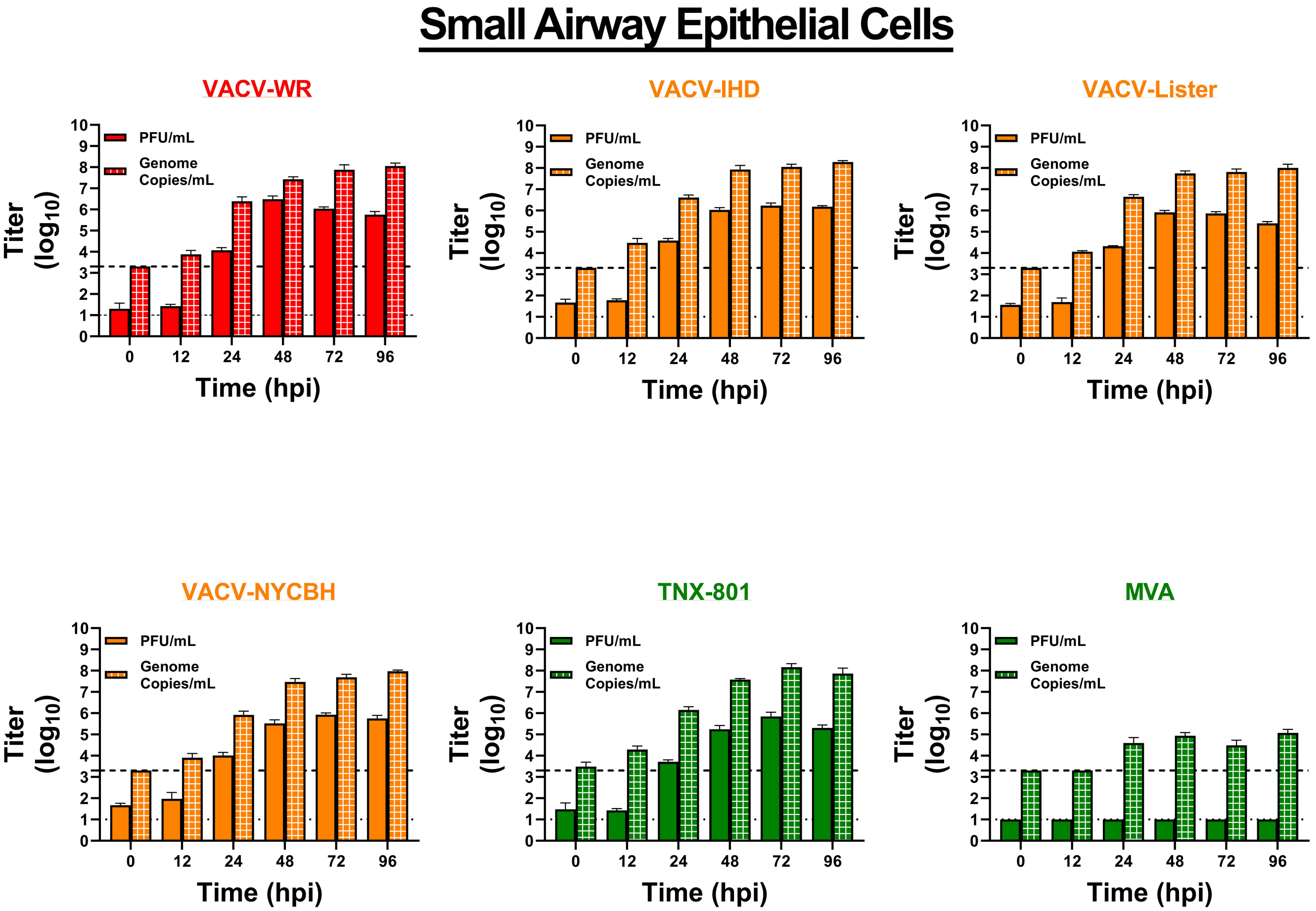

Supp. Figure 9

Lung Fibroblasts

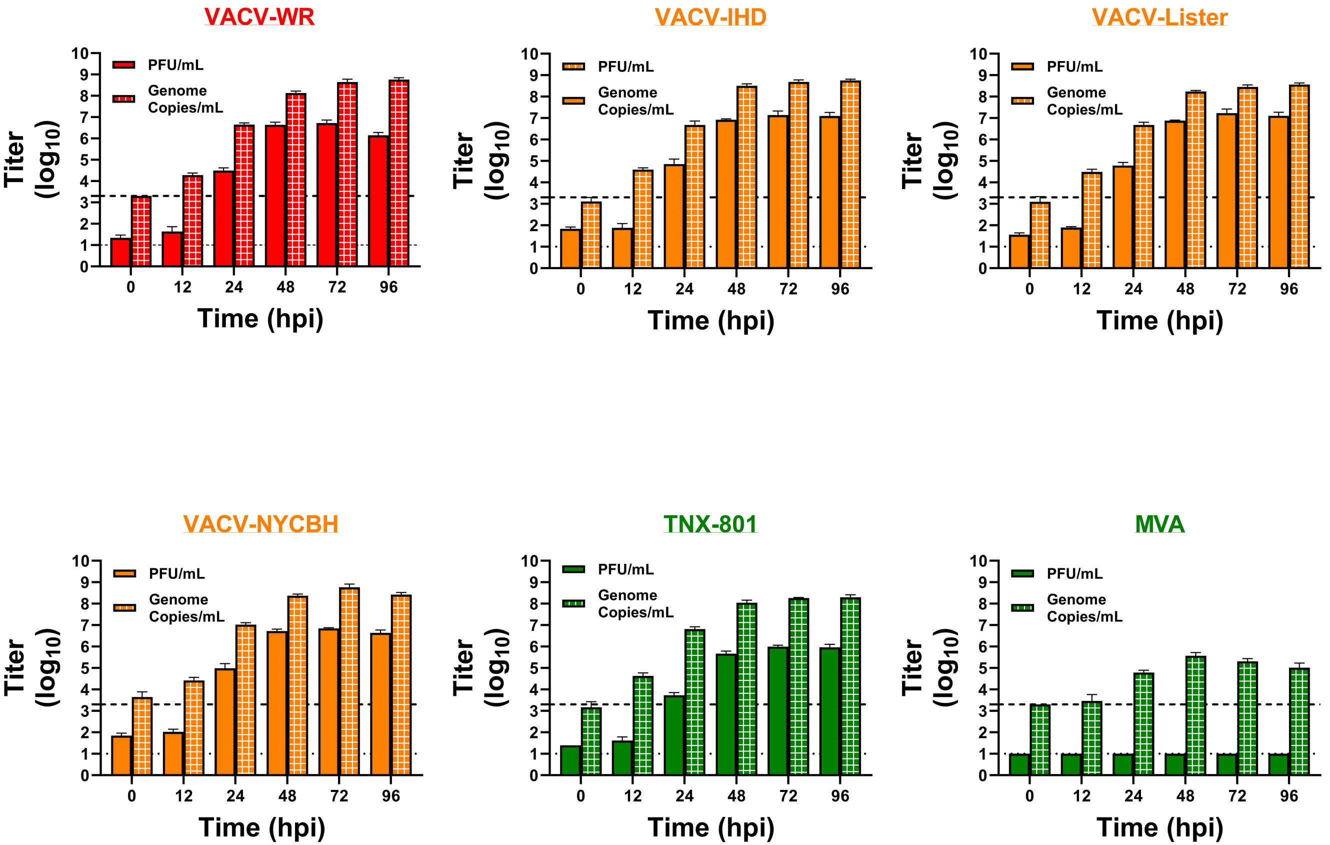

Supp. Figure 10

B-T Smooth Muscle Cells

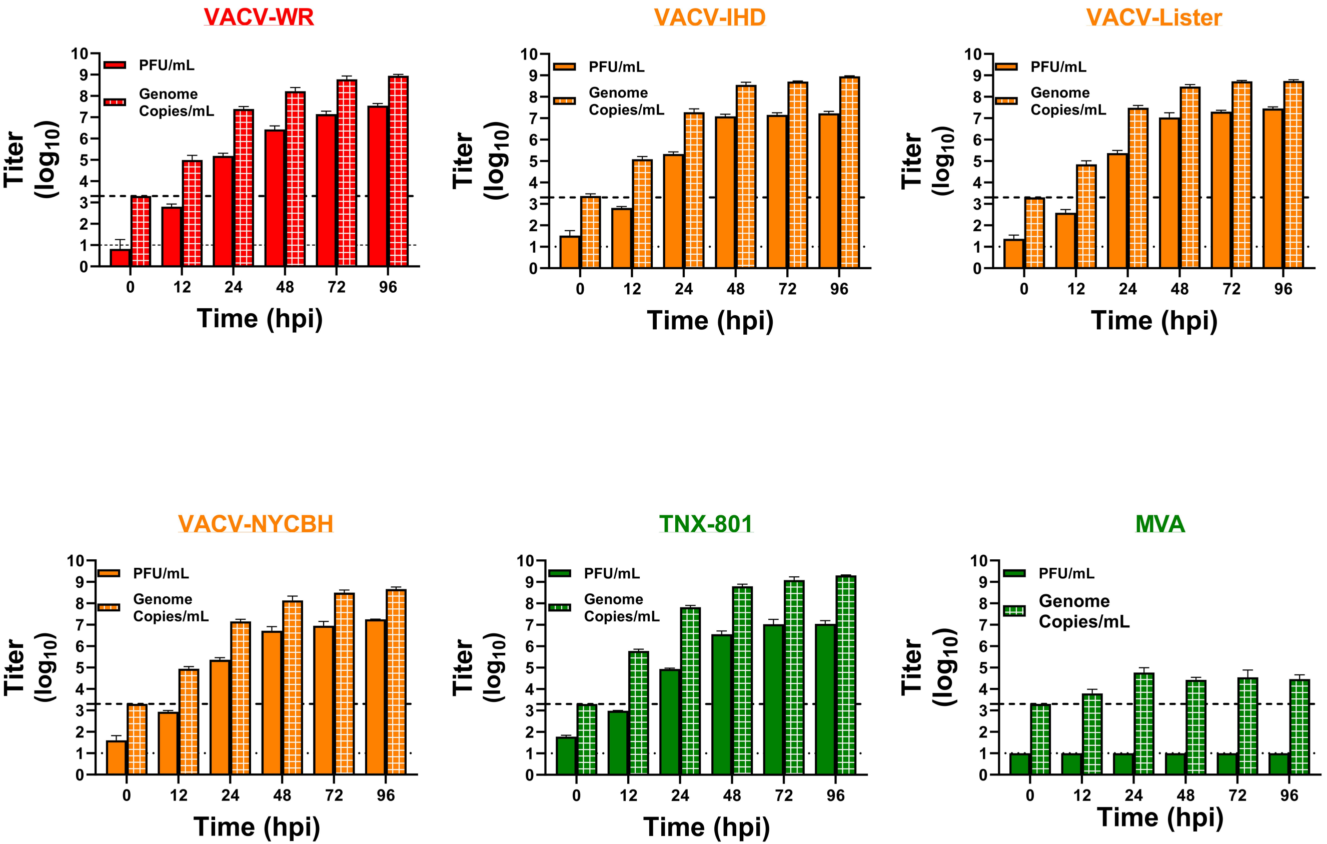

Supp. Figure 1. Comparison of infectious titers and genome copy of VACV strains, TNX-801, and MVA infection in BSC-40 cells. The limit of detection for plaque assay and genome copies are indicated via thin and thick dashed lines, respectively. The limit of detection is shown for the infectious (1.0 log_{10} PFU/mL) and qPCR (3.3 log_{10} genome copies per mL) assays with black dashed lines.

Supp. Figure 2. Comparison of infectious titers and genome copy of VACV strains, TNX-801, and MVA infection in Vero-E6 cells. The limit of detection for plaque assay and genome copies are indicated via thin and thick dashed lines, respectively. The limit of detection is shown for the infectious (1.0 log_{10} PFU/mL) and qPCR (3.3 log_{10} genome copies per mL) assays with black dashed lines.

Supp. Figure 3. Comparison of infectious titers and genome copy VACV strains, TNX-801, and MVA infection in human primary melanocytes. The limit of detection for plaque assay and genome copies are indicated via thin and thick dashed lines, respectively. The limit of detection is shown for the infectious (1.0 log_{10} PFU/mL) and qPCR (3.3 log_{10} genome copies per mL) assays with black dashed lines.

Supp. Figure 4. Comparison of infectious titers and genome copy of VACV strains, TNX-801, and MVA infection in human primary keratinocytes. The limit of detection for plaque assay and genome copies are indicated via thin and thick dashed lines,

respectively. The limit of detection is shown for the infectious (1.0 log_{10} PFU/mL) and qPCR (3.3 log_{10} genome copies per mL) assays with black dashed lines.

Supp. Figure 5. Comparison of infectious titers and genome copy of VACV strains, TNX-801, and MVA infection in human primary keratinocytes. The limit of detection for plaque assay and genome copies are indicated via thin and thick dashed lines, respectively. The limit of detection is shown for the infectious (1.0 log_{10} PFU/mL) and qPCR (3.3 log_{10} genome copies per mL) assays with black dashed lines.

Supp. Figure 5. Comparison of infectious titers and genome copy of VACV strains, TNX-801, and MVA infection in human primary dermal fibroblasts. The limit of detection for plaque assay and genome copies are indicated via thin and thick dashed lines, respectively. The limit of detection is shown for the infectious (1.0 log_{10} PFU/mL) and qPCR (3.3 log_{10} genome copies per mL) assays with black dashed lines.

Supp. Figure 6. Comparison of infectious titers and genome copy of VACV strains, TNX-801, and MVA infection in human primary skeletal muscle cells. The limit of detection for plaque assay and genome copies are indicated via thin and thick dashed lines, respectively. The limit of detection is shown for the infectious (1.0 log_{10} PFU/mL) and qPCR (3.3 log_{10} genome copies per mL) assays with black dashed lines.

Supp. Figure 7. Comparison of infectious titers and genome copy of VACV strains, TNX-801, and MVA infection in human primary bronchial-tracheal epithelial cells. The limit of detection for plaque assay and genome copies are indicated via thin and thick dashed lines, respectively. The limit of detection is shown for the infectious (1.0 $\log_{10}$ PFU/mL) and qPCR (3.3 $\log_{10}$ genome copies per mL) assays with black dashed lines.

Supp. Figure 8. Comparison of infectious titers and genome copy of VACV strains, TNX-801, and MVA infection in human primary small airway epithelial cells. The limit of detection for plaque assay and genome copies are indicated via thin and thick dashed lines, respectively. The limit of detection is shown for the infectious (1.0 $\log_{10}$ PFU/mL) and qPCR (3.3 $\log_{10}$ genome copies per mL) assays with black dashed lines.

Supp. Figure 9. Comparison of infectious titers and genome copy of VACV strains, TNX-801, and MVA infection in human primary lung fibroblasts. The limit of detection for plaque assay and genome copies are indicated via thin and thick dashed lines, respectively. The limit of detection is shown for the infectious (1.0 $\log_{10}$ PFU/mL) and qPCR (3.3 $\log_{10}$ genome copies per mL) assays with black dashed lines.

Supp. Figure 10. Comparison of infectious titers and genome copy of VACV strains, TNX-801, and MVA infection in human primary bronchial-tracheal smooth muscle cells. The limit of detection for plaque assay and genome copies are indicated via thin and thick dashed lines, respectively. The limit of detection is shown for the infectious (1.0

log_{10} PFU/mL) and qPCR (3.3 log_{10} genome copies per mL) assays with black dashed lines.
